# Identification of Essential Proteins Based on a New Combination of Local Interaction Density and Protein Complexes

**DOI:** 10.1371/journal.pone.0131418

**Published:** 2015-06-30

**Authors:** Jiawei Luo, Yi Qi

**Affiliations:** College of Computer Science and Electronic Engineering, Hunan University, Changsha, China; Semmelweis University, HUNGARY

## Abstract

**Background:**

Computational approaches aided by computer science have been used to predict essential proteins and are faster than expensive, time-consuming, laborious experimental approaches. However, the performance of such approaches is still poor, making practical applications of computational approaches difficult in some fields. Hence, the development of more suitable and efficient computing methods is necessary for identification of essential proteins.

**Method:**

In this paper, we propose a new method for predicting essential proteins in a protein interaction network, local interaction density combined with protein complexes (LIDC), based on statistical analyses of essential proteins and protein complexes. First, we introduce a new local topological centrality, local interaction density (LID), of the yeast PPI network; second, we discuss a new integration strategy for multiple bioinformatics. The LIDC method was then developed through a combination of LID and protein complex information based on our new integration strategy. The purpose of LIDC is discovery of important features of essential proteins with their neighbors in real protein complexes, thereby improving the efficiency of identification.

**Results:**

Experimental results based on three different PPI(protein-protein interaction) networks of *Saccharomyces cerevisiae* and *Escherichia coli* showed that LIDC outperformed classical topological centrality measures and some recent combinational methods. Moreover, when predicting MIPS datasets, the better improvement of performance obtained by LIDC is over all nine reference methods (i.e., DC, BC, NC, LID, PeC, CoEWC, WDC, ION, and UC).

**Conclusions:**

LIDC is more effective for the prediction of essential proteins than other recently developed methods.

## Introduction

Proteins are vital parts of living organisms, functioning as the main components of the physiological metabolic pathways of cells, and form densely connected modules to perform various biological functions. With the development of proteomics research in the post-genomic era, several protein-related topics, including discovery of the structures and functions of proteins, detection of interactions among proteins, and identification of essential proteins or protein complexes and functional modules, have become the major focus of many researchers. Essential proteins are those proteins that, if deleted, result in lethality or infertility [1]. Some recent results have suggested that comprehensive analyses of essential proteins will lead to a deeper understanding of the relationships between gene mutations and human diseases, thereby revealing the general principles of human diseases [2–4]. Therefore, the prediction of essential proteins has important theoretical and practice significance in biological and medical fields.

Generally, two types of methods are used to identify essential proteins. The first is experimental techniques, such as single gene knockout [5], RNA interference [6], and conditional gene knockout [7], all of which are time-consuming and expensive. The second is bioinformatics computational approaches, which are faster and less expensive than experimental techniques, as supported by the sharp increase in the amount of high-throughput data available. Many computational approaches have been proposed for identification of the correlations among the topological properties of a protein, protein interaction networks (PINs), and the essentiality of proteins.

As a new modality for the identification of essential proteins, computational approaches focus on the topological properties of biological networks according to the centrality-lethality rule [1], and graph theory has become the main theoretical basis for construction of most computational approaches. There are about two subclasses in computational approaches: (1) topological centrality measures at the network level, such as Li et al. have reported recently a topology potential-based method for identifying essential proteins and the basic idea is that each protein in a PIN can be viewed as a material particle which creates a potential field around itself and the interaction of all proteins forms a topological field over the network. By defining and computing the value of each protein’s topology potential, this method can obtain a more precise ranking which reflects the importance of proteins from the PPI network[8], and Tang et al. have proposed a cytoscape plugin, CytoNCA, for centrality analysis and evaluation of biological networks recently. CytoNCA supports eight different topological centrality measures and each can be applied to both weighted and unweighted biological networks[9], and (2) multi-information fusion measures, i.e., a combination of topological centrality measures and other biological information of proteins besides protein-protein interactions (PPIs), such as protein complexes [10, 11], gene ontology (GO) terms of proteins [12], gene expression data [13, 14], orthologous information[15], and overlapping essential modules[16]. However, the effectiveness of fusion strategies or mechanisms has not been sufficiently discussed. Therefore, it is critical to design suitable network-level methods integrated appropriately with biological information for prediction of essential proteins.

Existing results suggest that there is a correlation between essentiality and the degree of proteins in PINs, e.g., according to the centrality-lethality rule; this is also perhaps the main reason that most computational approaches must be sensitive to the topological structures of PINs. Additionally, subsequent experiments and analyses, e.g., yeast two-hybrid (Y2H) analyses, have shown that this type correlation may be too weak for binary or transient protein interactions [17, 18]. Moreover, Ryan et al. showed that entire complexes appear essential due to modular essentiality [19], and Wang et al. noted that larger protein complexes are more likely to be essential, explaining why essential genes are more likely to have high degrees of protein complex interactions [20]. Genes in humans whose protein products belong to the same protein complex are more likely to be associated with the same disease phenotype [21–23]. As shown in various studies, proteins tend to form densely connected modules with their adjacent nodes in order to perform specific biological functions, and essential proteins are the nodes in these modules that are required to maintain the existence of modules and biological functions [18]. In this sense, in addition to topological properties of proteins, we may also pay attention to the correlation between the essentiality of proteins and protein complexes in PINs. There are some results on discussing the relationship between the protein complexes and essentiality of proteins. Hart et al. have demonstrated a scoring method which may generate an integrated high-confidence subset of observed matrix-model interactions, and then it has been used to derive an accurate map of yeast complexes. Their results indicate that essentiality is a product of the protein complex rather than the individual protein[24]. Hart et al. focus on the protein complexes rather than essential proteins. Ren et al. have propose a centrality method, ECC, to identify essential proteins by integration of subgraph centrality in a PPI network and protein complexes information which is the sum of in-degree of protein *u* in a given protein complex sets. They have only discussed the ratio of a single essential protein in a given protein complexes[10]. Li et al. have reported recently another new centrality method, united complex centrality(UC), whose discussion on the relationship between essential proteins and protein complexes is similar to Ren et al[11]. Zhong et al. have constructed a method based on gene expression programming to predicting essential proteins combined by some centrality measures such as DC, BC and PeC etc[25]. We consider they have not analyzed the relationship among neighbors of essential proteins and those neighbors in a protein complex.

To overcome the limitations of existing computational approaches in current research on prediction of essential proteins, we have explored the interaction relationships among nodes in real protein complexes based on protein and protein interaction information, we partition proteins in a protein interaction network to two type nodes: interactive nodes and isolated nodes as described in section Definition of the problem and analyses. This kind of partition to proteins in complexes we proposed is different from the in-degree measure of one complex used by some existing studies mentioned above. And then we proposed a new identification method, local interaction density combined with protein complexes (LIDC), which is composed of three components. The first is a new centrality measure based on the local topological properties of proteins in protein complexes, called the local interaction density (LID); the second is the biological information of protein complexes, called the in-degree centrality of complex (IDC); and the third is a new integration strategy for combining LID and IDC. LID is expected to facilitate the identification of essential proteins from their neighbors, which interact with other neighbors. LIDC was applied to three PIN datasets of *Saccharomyces cerevisiae* and *Escherichia coli*, which are representative model organisms, downloaded from the DIP and MIPS databases.

Compared to nine conventional methods, i.e., degree centrality (DC) [1], betweenness centrality (BC) [26], Network Centrality (NC) [27], local interaction density (LID), PeC [13], co-expression weighted by clustering coefficient (CoEWC) [28], an iteration method for predicting essential proteins by integrating orthology with PPI network(ION)[15], weighted degree centrality (WDC) [29] and united complex centrality(UC)[11], of which DC, BC, and NC are well-known topological centrality measures and PeC, CoEWC, ION, WDC and UC are recent multi-information fusion measures that have shown good performance for protein identification, LIDC exhibited better performance for the identification of essential proteins based on comprehensive analyses of sensitivity, specificity, F-measures, positive predictive value, negative predictive value, accuracy, precision-recall curves and area under receiver operating characteristic (ROC) curve (AUC). In particular, when predicting MIPS datasets, the better improvement was clearly obtained by LIDC over all nine reference methods for every parameter measured.

## Materials and Methods

### Motivation

Most existing prediction methods are built on the centrality-lethality rule and the topological centrality of PINs; even recent multi-information fusion measures, which often perform better than purely topological centrality methods, usually include one type of topological centrality measure. To further improve the prediction capability of multi-information fusion measures, we may be confronted with two problems: 1) discovery of new topological centralities, and 2) development of more effective integration strategies.

With regard to existing topological centrality, we have learned that most high-degree hub nodes are not essential in yeast PINs [30]. The correlation between essentiality and topological centrality of single proteins in PINs may be weak for binary or transient protein interactions [18, 31]. On the other hand, proteins tend to form protein complexes with their adjacent nodes in order to perform specific biological functions. Hence, in this study, we explored the relationship between properties of proteins with their neighbors in real complexes and essentiality of proteins, rather than just considering the topological centrality of proteins at the global network level.

Through statistical analysis of the identification methods mentioned above, we noticed that some proteins have the same centrality score in a given ranking range predicted by one method, and these proteins may not be distinguished clearly, regardless of whether the protein is essential or nonessential. These limits may reduce the overall performance of existing identification methods. In order to achieve improved performance of identifying essential proteins, we even more present a new integration strategy that combines LID and information of protein complexes so as to construct LIDC, which achieved better performance in these ranking ranges: top 100, top 200, top 300, top 400, top 500 and top 600.

### Definition of the problem and analyses

A PIN is generally described as an undirected graph *G* = (*V*,*E*), which consists of a node set *V* and an edge set *E*. One node *v* ∈ *V* represents a unique protein, while one edge (*u*,*v*) ∈ *E* represents an interaction between protein *u* and protein *v*. The adjacency matrix is written as *A* of *G*, and the matrix (an *N × N* symmetric matrix) is written as *A* = *a*_*u*,*v*_, where the variable N is the total number of nodes in a PIN; thus,

$$a_{u,v}=\left\{ \begin{array}{l} 1,\;(u,v)\in E;\\ 0,\;\text{otherwise}. \end{array}\right.$$

**Fig 1:**
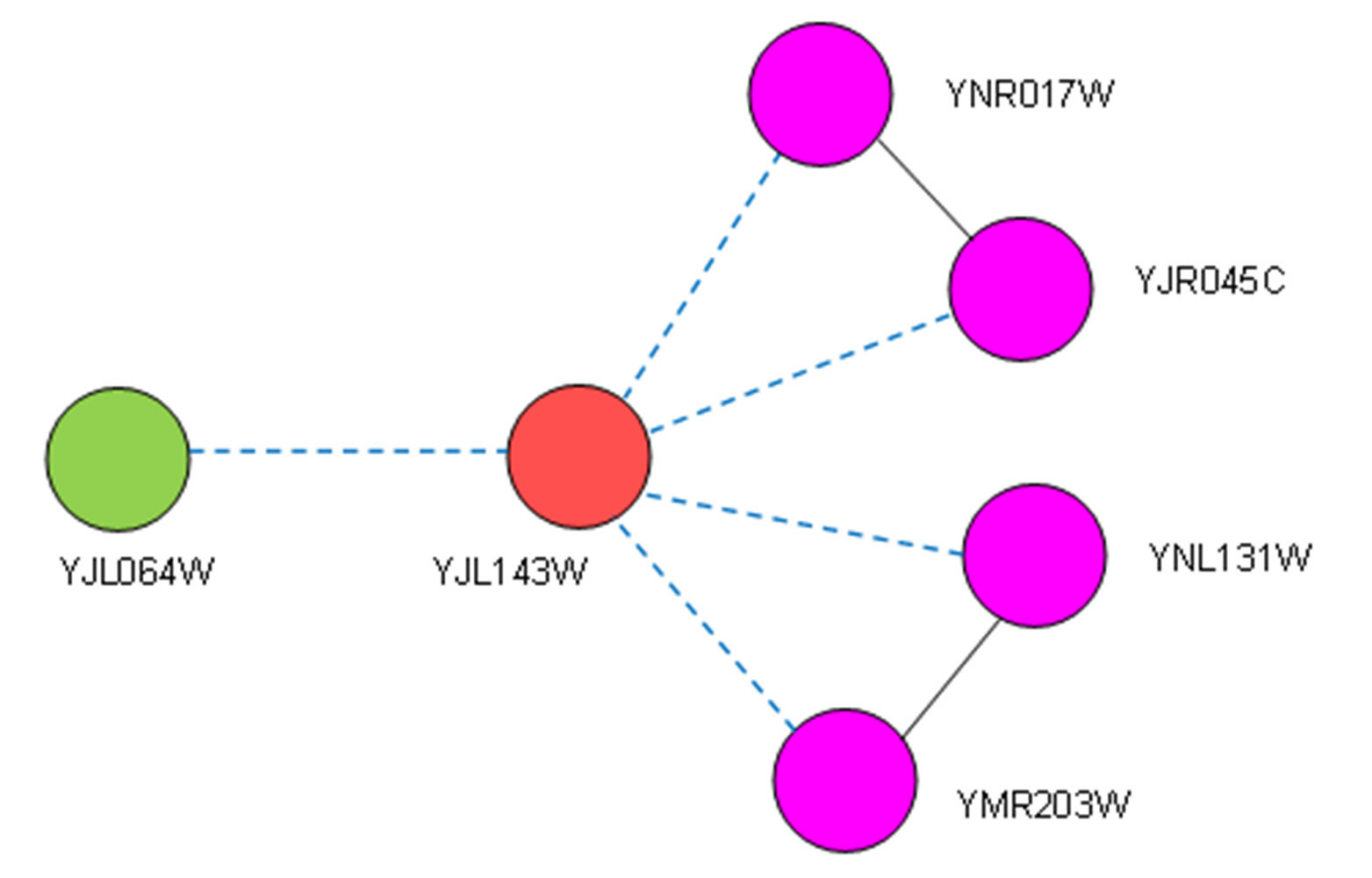
Protein YJL143W (red) is a no-star node with its five neighbors. VNB(u) = VNB_ISO(u)∪VNB_INT(u), u = YJL143W, VNB_INT(u) = {YNR017W,YJR045C, YNL131W,YMR203W}VNB_ISO(u) = {YJL064W}. Five blue long dashes represent the adjacent edges between YJL143W and its neighbors, and the two black solid lines represent interactive edges among interactive neighbors colored by purple. Green nodes are isolated neighbors, such as protein YJL064W. Protein YJL143W is an essential protein.

**Fig 2:**
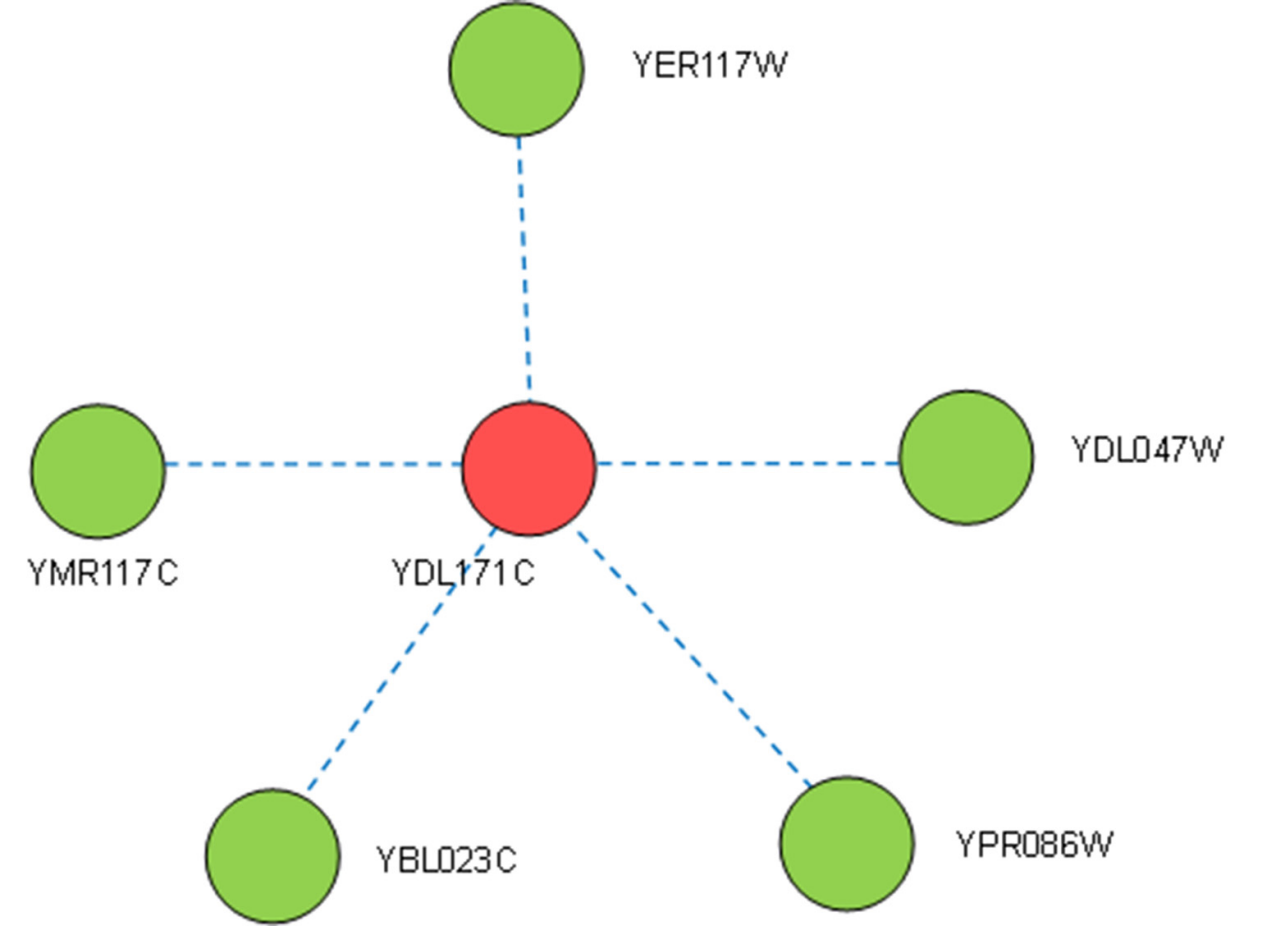
Protein YDL171C (red) is a star node with its five neighbors. The blue long dashes are the adjacent edges between the source node YDL171C and its green neighbors, which are completely isolated, meaning they will be isolated points in the adjacency subgraph of u if these adjacent edges can be ignored. Protein YDL171C is a nonessential protein.

*G*_NB_(*u*) = (*V*_NB_(*u*),*E*_NB_(*u*)) is the adjacency subgraph of a protein *u*, i.e., a source node *u* of *G*_NB_(*u*) in a PIN, where *V*_NB_(*u*) is the node set in *G*_NB_(*u*), and *E*_NB_(*u*) is the edge set in *G*_NB_(*u*). *V*_NB_(*u*) is the union of two neighbor node subsets, *V*_NB_ISO_(*u*) and *V*_NB_INT_(*u*), while *E*_NB_(*u*) is also the union of two neighbor edge subsets, *E*_NB_ADE_(*u*) and *E*_NB_INT_(*u*). Edges in *E*_NB_ADE_(*u*) are called adjacency edges and are interactions between a source node *u* and all of its neighbors; edges in *E*_NB_INT_(*u*) are called interactive edges and are those interactions among neighbor nodes of *u*. Nodes in *V*_NB_ISO_(*u*), called isolated neighbors, are nodes that only have adjacency edges to the source node *u*, while nodes in *V*_NB_INT_(*u*), called interactive neighbors, are nodes that have both adjacent edges to *u* and interactive edges among them, as shown in Fig 1. A node *u* is a star node if its *V*_NB_INT_(*u*) is empty and the topological structure of its adjacent subgraph is a star, e.g., protein YDL171C, as shown in Fig 2.

In order to analyze the correlations between interactive neighbors and essential proteins in terms of modularity, we first examined nodes in a real protein complex dataset based on our partition of nodes. In a yeast protein complex dataset containing 408 complexes and 1920 nodes, without considering the overlap among complexes [32] (whose interaction information was obtained from a yeast protein interaction network downloaded from the DIP database, which has 5616 proteins and 52833 interactions [33]), there were 140 essential proteins in 435 isolated neighbors whose in-degree in the complex was 1 (7% of 1920 nodes). Moreover, there were 1485 nodes which were interactive neighbors in complexes, with in-degrees of more than 1; 681 of these nodes represented essential proteins (35% of 1920 nodes). Additionally, according to degree analysis of nodes in the yeast PIN mentioned above, there were 2705 star nodes, of which only 13.1% were essential proteins, and 2388 non-star nodes (indicating the presence of some interactive neighbors), out of which 34% were essential proteins, e.g., protein YJL143W in Fig 1. Therefore, we proposed a new local topological measure, LID, based on protein complexes, to determine the essentiality of a protein by evaluating the relationship between a protein and its interactive neighbors.

### New identification algorithm: LIDC

LIDC is a multi-information fusion measure proposed in this paper that is composed of three parts as follows.

#### 1) A new topological centrality: LID centrality

The LID of a node *u* (LID(*u*)) is defined as the density of interactions among its interactive neighbors:

$$\text{LID}(u)=\frac{\left|E_{\text{NB\_INT}}(u)\right|}{\left|V_{\text{NB\_INT}}(u)\right|}$$ (1)

where the operator || is a count of the number of elements in a set. For example, in Fig 1, node *u* is protein YJL143W, and we then have |*E*_NB_INT_(*u*)| = 2 and |*V*_NB_INT_(*u*)| = 4, such that LID(*u*) = 0.5. Obviously, LID(*u*) will be larger if there are fewer neighbors of node *u*, resulting in denser interactions among the nodes.

#### 2) IDC

Protein complexes are stable macromolecular assemblies that perform many diverse biochemical activities essential to cell homeostasis, growth, and proliferation; proteins within these complexes appear at the same time and in the same location, and more some proteins participate in more than one complex. At the network level, protein complexes are usually substructures in PINs. In our design, we may compute the IDC of a protein to identify the essential proteins. The IDC of a protein *u* in a protein complex set is defined as:

$$\text{IDC}(u)=\sum_{i\in ComplexSet(u)}\text{IN-Degree}{\left(u\right)}_{i}$$ (2)

where ComplexSet(*u*) denotes a set of protein complexes that include protein *u*, and IN-Degree(*u*)_*i*_ is the in-degree value of protein *u* in *i*th protein complex that belongs to ComplexSet(*u*). IN-Degree(*u*)_*i*_ is computed as:

$$\text{IN-Degree}{\left(u\right)}_{i}=DC{\left(u\right)}_{i}$$ (3)

where DC(*u*)_*i*_ is the degree value of protein *u* in complex *i*, which is *i*th element in ComplexSet(*u*).

#### 3) Integration strategy of LID and IDC

The purpose of our new integration strategy is to overcome the constraints of existing identification methods, such as existing identification methods lack sufficient flexibility to adapt to various network structures of PINs. The construction of topological centrality measures is based on a special dataset, generally becoming the innate deficiency in multiple datasets, indicating that the performance of this type of identification method is sensitive to the structures of PINs. There are two types of integration strategies used to combine multi-information fusion measures. The first is that the prediction score of proteins is the product of values of multifeature information, such as PeC, CoEWC and UC, and the second is that the predicting score of proteins is the linear sum of values with some coefficients of multifeature information, such as ION and WDC. The way of product increases the network structure dependence of those methods than the linear sum way without sufficient guarantee of the quality of the dataset, leading to high false positives and false negatives of interactions among proteins. On the other hand, the pattern of the linear sum contains some coefficients that adjust the proportions of feature values for each type of information in the predicting score of the proteins, and however many of these coefficients are constants, which, in most cases, depend on a given PIN, thereby increasing the network structure sensitivities of these methods as well. Therefore, we consider a new integration strategy to pursue good performance in all six ranking ranges at the same time in order to improve flexibility in the context of multiple PINs.

The new integration strategy we consider here has the advantages of the topological centrality measure LID and the prediction method IDC. Let RANK(*u*) be the order number of the descending sort of protein *u* according to LID(*u*) in a PIN. According to the modularity of proteins and the integrating strategy, there are four cases to show some biological meanings of our LIDC. The first case is when LID(*u*) is larger and IDC(*u*) is larger, LIDC(*u*) is also larger which means neighbors of protein *u* have more interactions among them so that protein *u* and its neighbors have more modularity and the probability of protein *u* be an essential protein also is increased. The second case is when LID(*u*) is smaller and IDC(*u*) is smaller, LIDC(*u*) is smaller which means neighbors of protein *u* have fewer interactions among them so that protein *u* and its neighbors have smaller modularity and the probability of protein *u* be an essential protein is decreased. The third case is when LID(*u*) is larger and IDC(*u*) is smaller, LIDC(*u*) mainly depends on LID values which means we maybe predict some new protein complexes around protein *u* from the perspective of biology. The forth case is when LID(*u*) is smaller and IDC(*u*) is larger, LIDC(*u*) depends on IDC values which means we maybe find out some new interactions among protein *u* and its neighbors.

On the other hand, LIDC with our new integrating strategy is a more soft computing method than those integrating ways with fixed coefficient style for multi information used in PeC, ION, WDC and UC because RANK(*u*) is the order number of the descending sort of protein *u* according to LID(*u*) depended on a given PIN, which means RANK(*u*) will be changed with different PPI networks. The flexibility of our new integrating strategy will improve the expansibility of LIDC.

### LIDC algorithm for identification of essential proteins

After summarizing and analyzing the experimental results of most topological centrality measures, including our new centrality LID, we found that there was no single centrality measure that could predict all essential proteins correctly and completely in a given PIN. Therefore, we should construct multi-information fusion measures in order to improve the performance of identification synthetically. Thus, we chose the IDC as our second biological information source in our new identification method because of the modularity of protein function, as mentioned above. Therefore, the LIDC of protein *u* can be computed as:

$$\text{LIDC}(u)=LID(u)\times (1-\frac{RANK(u)}{N})+IDC(u)\times \frac{RANK(u)}{N}$$ (4)

where LID(*u*) is the value of the LID, IDC(*u*) is the value of IDC of the protein complex of protein *u*, and *N* is the number of proteins in the current PIN. RANK(*u*) is the order number of the descending sort of protein *u* according to LID(*u*) in the current PIN, which can vary with different datasets such that LIDC can be more flexible and adjustable than traditional, simple, empirical, single-parameter patterns.

From the definition and analyses of LIDC above, LIDC may identify essential proteins more effectively than existing identification methods.

## Results and Discussion

### Experimental dataset

To evaluate the performance of our proposed LIDC method, we considered PPI data of *Saccharomyces cerevisiae* (yeast) as one of experimental materials because this model organism has relative complete, reliable PPI and essential protein data. The PPI network of *Saccharomyces cerevisiae* was downloaded from two databases: the DIP database [33](S1 Text), and the MIPS database [34] (S2 Text). A collection of essential proteins of *S*. *cerevisiae* was gathered from several databases, including MIPS [34], SGD [35], DEG [36], and SGDP [37], yielding 1285 essential proteins and 4394 nonessential proteins (S1 Excel).

In this study, we integrated four real protein complex sets (CM270, CM425, CYC408, and CYC428) into one comprehensive protein complex set. Of the four protein complexes datasets, only complexes with a size of more than two nodes were kept. CM270 is the gold standard protein complex set and was downloaded from the MIPS database [34]. It contains 270 complexes and 1230 proteins. The second real complex set (CM425), described by Friedel et al [38], was integrated from three sources, i.e., MIPS [34], Aloy et al [39], and the SGD database [35], and contains 425 complexes and 1970 proteins. The third and fourth complex sets (CYC408 and CYC428) were obtained from CYC2008 of the Wodak Laboratory [32, 40]. We combined these four protein complex datasets to obtain a new complex dataset containing 745 protein complexes (comprised of 2167 proteins); we called this dataset Complex_745 (S2 Excel). Note that data for these protein complexes were detected through experimental methods and thus did not contain all proteins within the given PPI networks, which means an IDC score of protein *u* is zero if protein *u* does not belong to any known protein complexes. This is an inherent defect of IDC that limits the use of IDC as a stand-alone independent identification method because the number of proteins in these protein complexes are less than those in ppi data and it may not be enough to detect essential proteins in a protein interaction network only through IDC, meanwhile essential proteins are more likely to be members of protein complexes so that the IDC is informative mentioned above. Thus we design a multi information fusion method LIDC and want to improve the performance of prediction for essential proteins based on LID and IDC together.

Next, we constructed two yeast PINs firstly for evaluating LIDC from two PPI datasets whose nodes represented proteins and edges represented interactions among proteins. The first network downloaded from the DIP database, marked YDIP_5093, included 5093 proteins and 24743 interactions. Its average degree was about 9.72. The second network downloaded from the MIPS database, marked YMIPS_4546, included 4546 proteins and 12319 interactions. Its average degree was about 5.42. The numbers of interactions in the two PINs were counted after removing self-interactions and duplicate interactions. With regard to the essentiality of proteins in a PIN, nodes were grouped into three subsets in a network: essential proteins, nonessential proteins, and unknown proteins covered by neither the essential protein set nor the nonessential protein set. We incorporated the unknown protein set into the nonessential protein set for the sake of easier discussion in this paper. There were 1167 essential proteins and 3926 nonessential proteins in network YDIP_5093 and 1016 essential proteins and 3530 nonessential proteins in network YMIPS_4546. The proportion of essential proteins in each PIN was nearly equal, which helped us to examine the precision of LIDC rationally. The details of nodes within the two PINs are shown in Table 1.

**Table 1:** The two yeast protein-protein interaction networks obtained from two PPI datasets (DIP and MIPS).

Data sets	Data sources	Proteins	Interactions	Essential proteins	Non-essential proteins	Unknown proteins
**YDIP_5093**	DIP	5093	24743	1167	3591	335
**YMIPS_4546**	MIPS	4546	12319	1016	3195	335

The global characteristics of the two PINs were similar for most items in Table 2, except for the average degree, which was 9.72 in YDIP_5093 and 5.42 in YMIPS_4546 (i.e., 1.79 times higher in YDIP_5093 than in YMIPS_4546), suggesting that YMIPS_4546 may be more sparse and more difficult to use for prediction than YDIP_5093.

**Table 2:** The global characteristics of two yeast protein interaction networks.

Data sets	Max degree	Average degree	Heterogeneity of degree	Nodes in max connected component	Average distance in max connected component
**YDIP_5093**	280	9.72	4.01	5052	3.84
**YMIPS_4546**	286	5.42	5.91	4385	4.42

*Heterogeneity(v) = average(v* ^*2*^ *)/(average(v))* ^*2*^

From local topological characteristics of PINs shown in Table 3, we found that average values of interactive edges, interactive neighbors, and isolated neighbors in YDIP_5093 were roughly two times higher than those in YMIPS_4546, while the heterogeneities of interactive edges, interactive neighbors, and isolated neighbors in YMIPS_4546 were 2–4 times higher than those in YDIP_5093. The heterogeneity of interactive edges was 42.64 in YMIPS_4546, indicating that the distribution of the interactive edges of nodes was quite unbalanced in this PIN. These heterogeneities of interactive edges suggested that it was not easy for LIDC to achieve better performance in YMIPS_4546.

**Table 3:** The local characteristics of two yeast protein interaction networks.

Data sets	Average interactive edges	Average interactive neighbors	Average isolated neighbors	Heterogeneity of interactive edges	Heterogeneity of interactive neighbors	Heterogeneity of isolated neighbors
**YDIP_5093**	10.75	5.45	4.27	10.52	5.85	3.77
**YMIPS_4546**	6.76	2.48	2.93	42.64	12.28	6.18

### Evaluation methods

In general to evaluate the overall performance of LIDC, we first ranked the results of LIDC in descending order and then chose proteins in six different ranking ranges (top 100–600) as essential protein candidate sets of the two PINs. The more essential proteins were identified in each potential set for the given method, the better the performance of the method.

And then for assessing the effectiveness and accuracy of LIDC, we selected the top 20% of ranking results from LIDC which were regarded as an essential protein candidate sets, and the rest part of the results which were considered nonessential protein sets for computing some statistical results. We then calculated several parameters, including the values of true positives (TPs), true negatives (TNs), false positives (FPs), false negatives (FNs), and obtained the outcomes of six common statistical methods using these values (TP, TN, FP, and FN), which are usually used to evaluate the effectiveness of an algorithm. Sensitivity was defined as *SN* = *TP* / (*TP* + *FN*); specificity was defined as *SP* = *TN* / (*TN* + *FP*); positive predictive value (PPV) was defined as *PPV* = *TP* / (*TP* + *FP*); negative predictive value (NPV) was defined as *NPV* = *TN* / (*TN* + *FN*); F-measure was defined as *F* = 2 × *SN* × *PPV* / (*SN* + *PPV*); accuracy was the proportion of correct prediction results of all the results, which is defined as *ACC* = (*TP* + *TN*) / (*Posi* + *Nega*), where *Posi* and *Nega* are the total number of essential proteins and nonessential proteins, respectively. The precision-recall curve has been finished in which Precision = *TP* / (*TP* + *FP*) and Recall = *TP* / (*TP* + *FN*). In addition, identification of essential proteins was also considered a classification problem; therefore, the area under the receiver operating characteristic (ROC) curve (AUC) was a suitable measure used to estimate the performance of our new method in which TPR = *TP* / (*TP* + *FN*) and FPR = *FP* / (*FP* + *TN*), and then may be computed through trapezoidal integration methods.

### Comparison of LIDC with other centrality measures

In order to validate the performance of LIDC, a comparison was carried out with nine classical identification measures: DC, BC, NC, LID, PeC, CoEWC, WDC, ION and UC. Among these, DC and BC are classic local and global topological centrality measures, respectively, and have been used by many researchers in various fields [1, 13, 26]. Additionally, NC is a core measure used for some multi-information fusion measures and a good local topological centrality measure [13, 27–29]. PeC was recently reported in 2012 and is based on the integration of PPI data and gene expression data. Moreover, PeC outperforms 15 other centrality measures in the yeast PPI network [13], including DC. CoEWC, WDC, ION and UC are two of the most recently reported multi-information fusion measures. ION proposed in 2012 shows the orthologous information of proteins is effective to detect essential proteins. CoEWC in 2013 may capture the properties of both data hubs and party hubs despite that the two hubs have very different clustering properties, meanwhile WDC, proposed in 2014, is used to show the linear correlation of topological centrality and relevant gene expression data. UC reported in 2015 is a combination of NC and in-degree of protein complexes.

**Fig 3:**
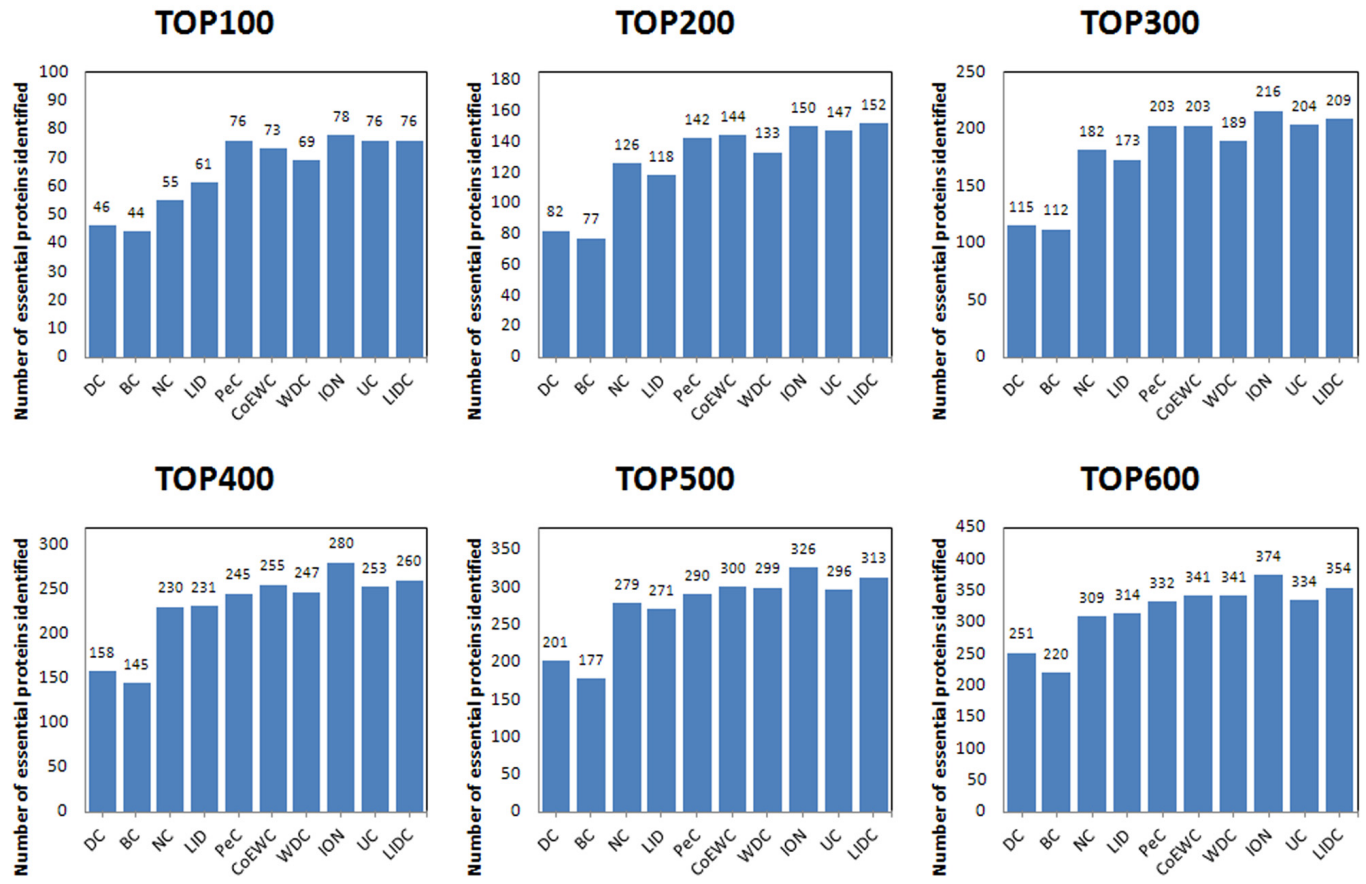
Comparison of the number of essential proteins from the top 100–600 identified by LIDC and nine other prediction measures in the YDIP_5093 PIN.

**Fig 4:**
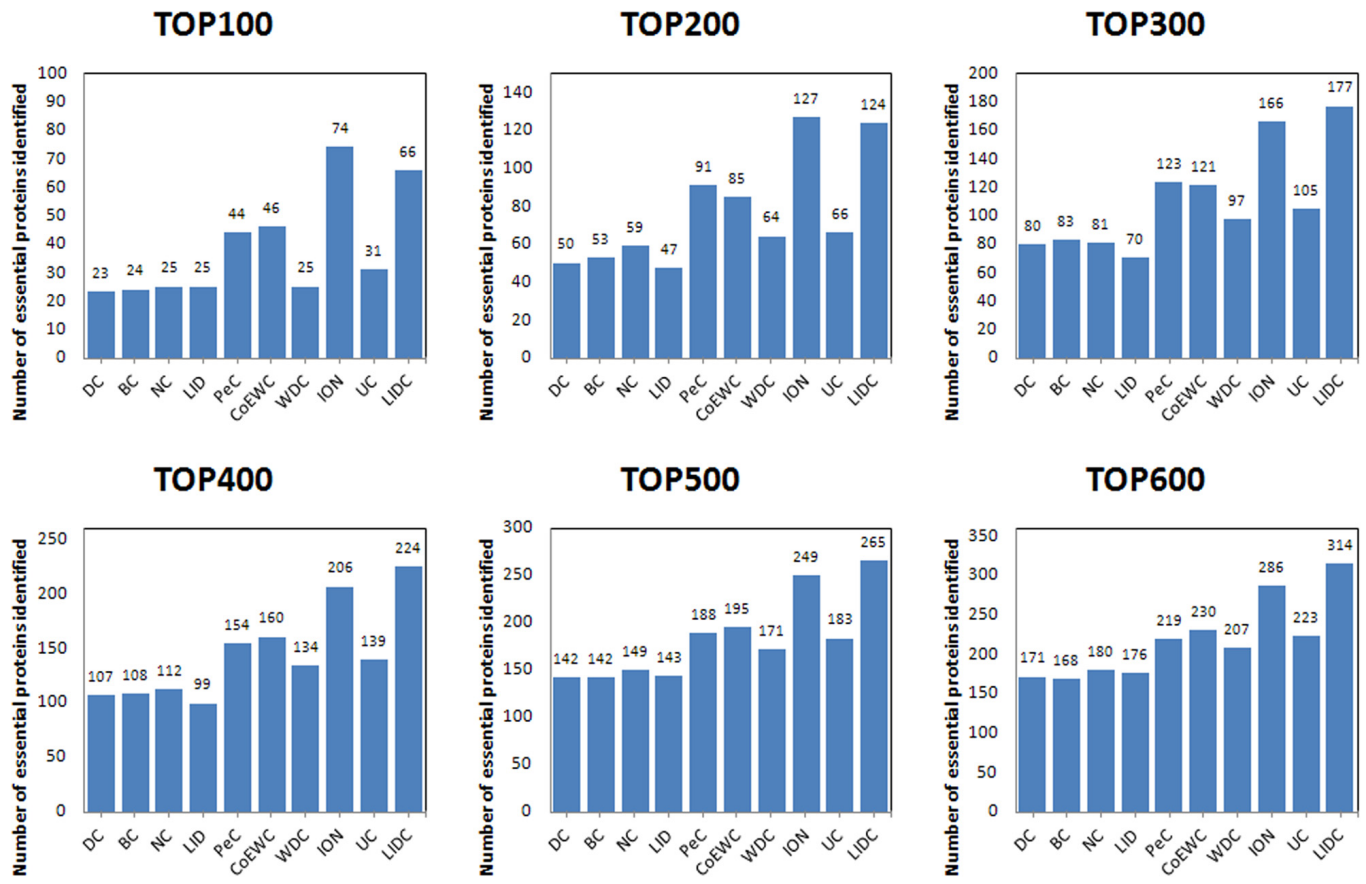
Comparison of the number of essential proteins from the top 100–600 identified by LIDC and nine other prediction measures in the YMIPS_4546 PIN.

For the purpose of performance testing of LIDC after ranking proteins in descending order based on their LIDC values and other methods, the six ranking ranges (from the top 100 to the top 600) were chosen as essential candidates, and finally, the essentiality of the proteins was checked through the collection of essential proteins described in the *Experimental dataset* section. The numbers of essential proteins predicted by LIDC and the other nine methods in the two yeast networks are shown in Figs 3–4; Fig 3 reported the results for the top 100–600 in YDIP_5093 while Fig 4 reported the results in YMIPS_4546.

As shown in Fig 3, the performances of LIDC were better than eight other methods for identifying essential proteins except ION in six comparisons from the top 100 to the top 600 in dataset YDIP_5093. Totally, LIDC is the second best method in this dataset, meanwhile the results of LIDC may oppose more modularity than all nine reference methods as showed in section Assessment of the modularity of proteins predicted by LIDC and six other methods. And this better modularity character of results has confirmed the rationality of LIDC and the consistency of our design purpose of this prediction method described in front sections of the this article.

As shown in Fig 4, the performance of LIDC was better than nine other methods for identifying essential proteins for six comparisons from the top 100 to the top 600 ranges in dataset YMIPS_4546. In particular, LIDC yielded a better improvement over the best results of the nine other methods for predicting from top 300 to top 600 (Table 4). Thus, experiments indicated that LIDC could identify more essential proteins than reference methods.

**Table 4:** Statistical analyses of improvement according to comparisons between LIDC and the best results of the nine other methods in each ranking range.

Ranking range	Best result of the other seven methods	LIDC	Values of Improvement	Proportion of Improvement
**TOP100**	74	**66**	-8	-10%
**TOP200**	127	**124**	-3	-2%
**TOP300**	166	**177**	11	7%
**TOP400**	206	**224**	18	9%
**TOP500**	249	**265**	16	6%
**TOP600**	286	**314**	28	10%

The results are in YMIPS_4546 PIN.

### Validated by jackknife methodology

**Fig 5:**
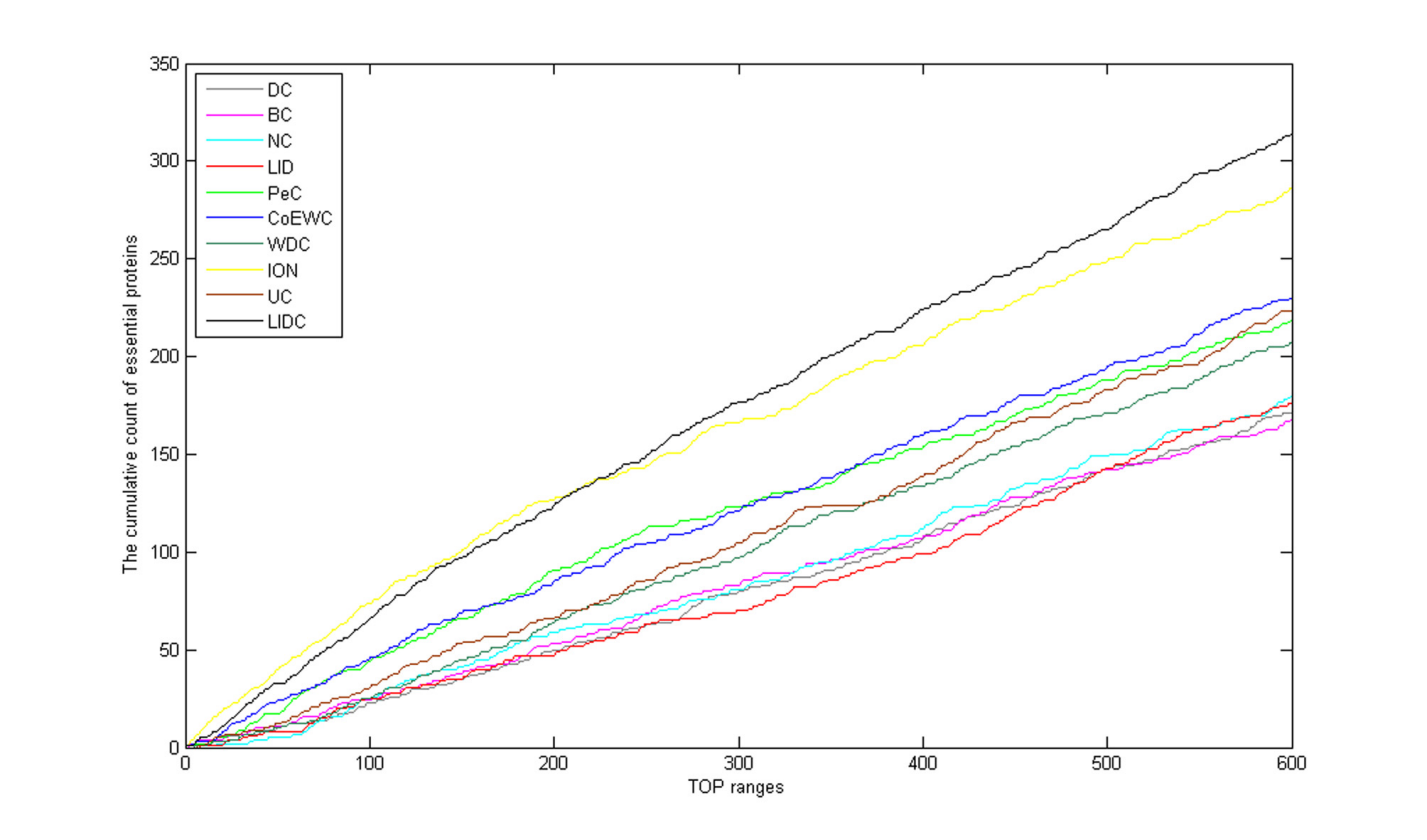
Comparison results by a jackknife methodology among LIDC and nine other prediction measures in the YMIPS_4546 PIN.

The jackknife methodology [41] has been used to compare the performance of LIDC with other nine previously proposed centrality measures (DC, BC, NC, LID, PeC, CoEWC, WDC, ION and UC) in YMIPS_4546 PIN. The comparison results are shown in Fig 5. In Fig 5, proteins are ordered from the highest value to the lowest value for each centrality measure whose top 20% have been selected from the ranking results were regarded as the essential protein candidate set, and then the cumulative counts of essential proteins are shown. It is clear that the curve of LIDC is higher than all nine reference measures from top 220 to top 600. This shows us that LIDC is more effective for detecting essential proteins.

### Verification of six statistical methods and PR curves

**Fig 6:**
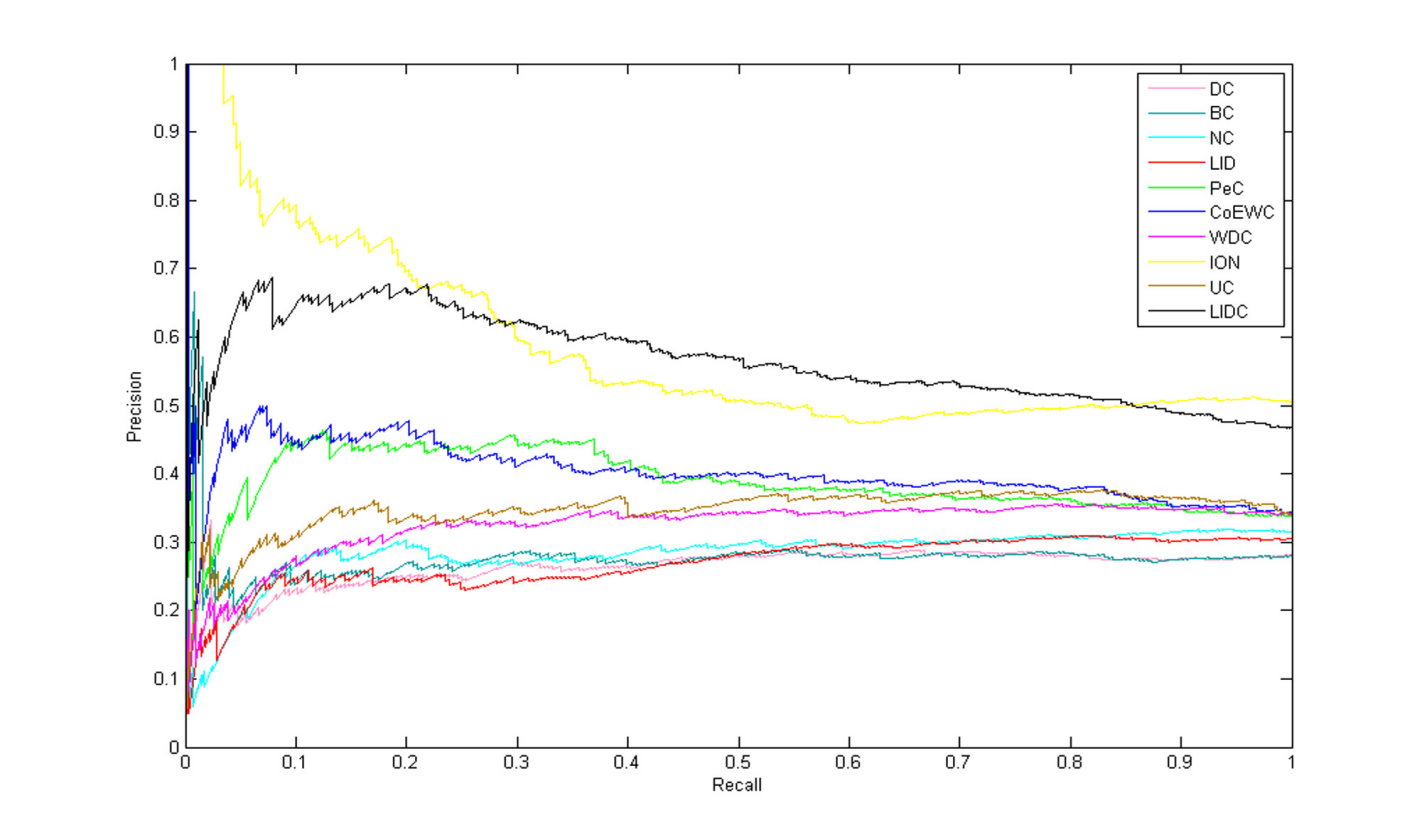
Comparison results by precision-recall(PR) curves among LIDC and nine other prediction measures in the YMIPS_4546 PIN.

In order to evaluate the overall performance of LIDC synthetically, we took advantage of six statistical analysis parameters, including sensitivity, specificity, PPV, NPV, F-measure, and accuracy. The top 20% selected from the ranking results of all methods was regarded as the essential protein candidate set, while the remaining results were considered the nonessential protein set (1019 and 909 proteins, respectively, for the two PINs). The sensitivity, specificity, PPV, NPV, F-measure, and accuracy of LIDC were higher than those of the eight other methods for both PINs, especially in YMIPS_4546 PIN (Table 5), and roughly similar to the results of ION, in which the differences about six statistical methods between LIDC and ION are small. These data indicated that LIDC could identify essential proteins more accurately than the other eight tested methods except ION and the same conclusion is also obtained by precision-recall curves among LIDC and nine other reference measures as shown in Fig 6. Moreover the precision and recall is a pair of paradoxes generally and a kind of mutual restraints each other. We consider that a method should have a more stable performance if the trend of its precision-recall curve may be more steady. As shown in Fig 6, the trend of LIDC’s PR curve is more steady than that of ION, and then indicates that LIDC may be oppose a more stable performance. Based on the analyses of tops, six statistical methods, precision-recall curves, rocs and aucs, we consider the performance of LIDC is better than that of ION synthetically.

**Table 5:** Comparison of the sensitivity (SN), specificity (SP), positive predictive value (PPV), negative predictive value (NPV), F-measure (F), and accuracy (ACC) between LIDC and the nine other prediction methods for the top 20% of proteins in two different PINs.

Data sets	Methods	SN	SP	PPV	NPV	F-measure	ACC
**YDIP_5093**	DC	0.35	0.85	0.41	0.81	0.50	0.73
	BC	0.31	0.83	0.35	0.80	0.45	0.71
	NC	0.40	0.86	0.46	0.83	0.54	0.75
	LID	0.39	0.86	0.45	0.83	0.54	0.75
	PeC	0.40	0.86	0.46	0.83	0.55	0.75
	CoEWC	0.41	0.86	0.47	0.83	0.56	0.76
	WDC	0.42	0.86	0.48	0.83	0.56	0.76
	ION	0.41	0.86	0.53	0.84	0.61	0.78
	UC	0.42	0.86	0.48	0.83	0.57	0.76
	LIDC	**0.44**	**0.87**	**0.50**	**0.84**	**0.59**	**0.77**
**YMIPS_4546**	DC	0.25	0.82	0.28	0.79	0.38	0.69
	BC	0.25	0.81	0.28	0.79	0.38	0.69
	NC	0.28	0.82	0.31	0.80	0.42	0.70
	LID	0.27	0.82	0.30	0.80	0.41	0.70
	PeC	0.30	0.83	0.34	0.80	0.44	0.71
	CoEWC	0.31	0.83	0.34	0.81	0.45	0.71
	WDC	0.30	0.83	0.34	0.81	0.45	0.71
	ION	0.45	0.86	0.49	0.84	0.59	0.77
	UC	0.31	0.83	0.34	0.81	0.45	0.71
	LIDC	**0.42**	**0.86**	**0.46**	**0.84**	**0.56**	**0.76**

### Validation by the ROC curve and AUC

**Fig 7:**
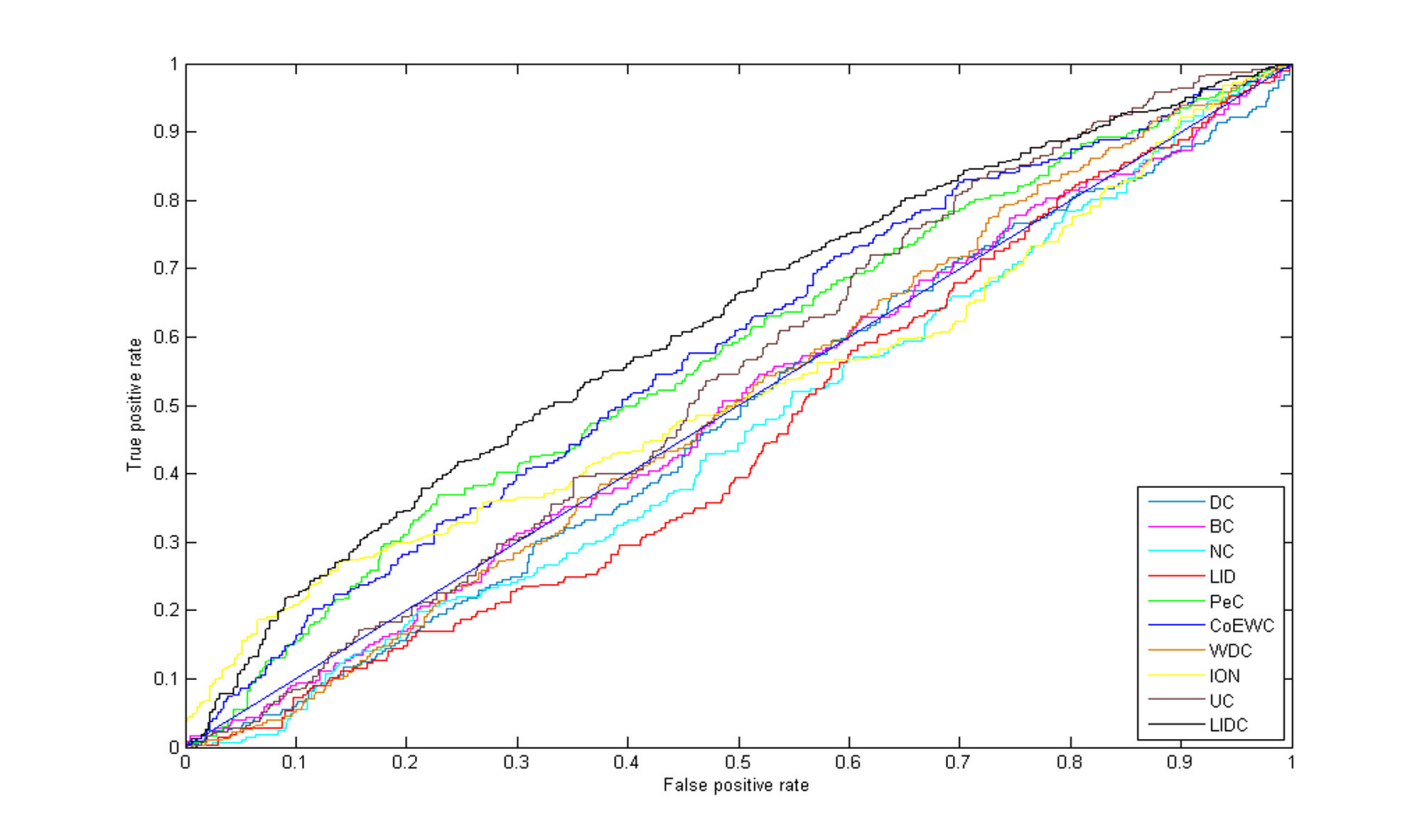
Comparison results by ROC curves among LIDC and nine other prediction measures in the YMIPS_4546 PIN.

The identification of essential proteins may be considered a two-class classification problem, rather an approximate imbalanced classification problem because there are usually two or three more nonessential proteins than essential proteins in PINs. For example, there were 1167 essential proteins and 3926 nonessential proteins in YDIP_5093 and 1016 essential proteins and 3530 nonessential proteins in YMIPS_4546. Hence, AUC is a suitable measure for estimating the efficiency of classifications between LIDC and other reference methods. The top 20% selected from the ranking results of all methods was regarded as the essential protein candidate set, while the remaining results were considered the nonessential protein set (1019 and 909 proteins, respectively, for the two PINs). The AUC of LIDC was better than those of nine reference methods in the YMIPS_4546 PINs (0.62 for YMIPS_4546), while 0.66 for YDIP_5093 is very close to the best AUC(0.67) of those results in other reference measures. The performance of LIDC for this imbalanced two-class classification for identification of essential proteins was best in YMIPS_4546 PIN as shown in Table 6, and the same conclusion is obtained by ROC curves among LIDC and nine other reference measures as shown in Fig 7.

**Table 6:** Comparisons of AUCs between LIDC and the nine other methods for the top 20% of proteins in two different yeast PINs.

Methods	YDIP_5093	YMIPS_4546
**DC**	0.51	0.49
**BC**	0.52	0.50
**NC**	0.61	0.46
**LID**	0.62	0.45
**PeC**	0.67	0.57
**CoEWC**	0.66	0.58
**WDC**	0.64	0.50
**ION**	0.65	0.52
**UC**	0.67	0.54
**LIDC**	**0.66**	**0.62**

### Effects of the new integration strategy

To assess the effects synthetically of the new integration strategy on LIDC in YDIP_5093 and YMIPS_4546 PINs, we analyzed the relative increasing ratio as a measure for evaluating performance, using LID as the reference method in 12 rank ranges top 100–600 and top 1%-25%. LID is a topological centrality measure whose performance was poorer than NC, a core component in PeC, CoEWC, and WDC, of the six comparisons for each PIN, as shown in Figs 3–4. The relative increasing ratio represented the ratio of the increase of identification method results between two adjacent ranking ranges and the increase of these two adjacent ranking ranges; for example, the relative increasing ratio between the top 1% and top 100 of LID in YDIP_5093 was 31/49 = 0.63, where 31 is the increase in two successive prediction results from the top 1% to top 100, and the denominator 49 is the increase in two successive ranking ranges (top 1% = 51 to top 100). We propose that the relative increasing ratio may describe changes in the performance improvement of methods at each ranking range compared to the former range.

**Fig 8:**
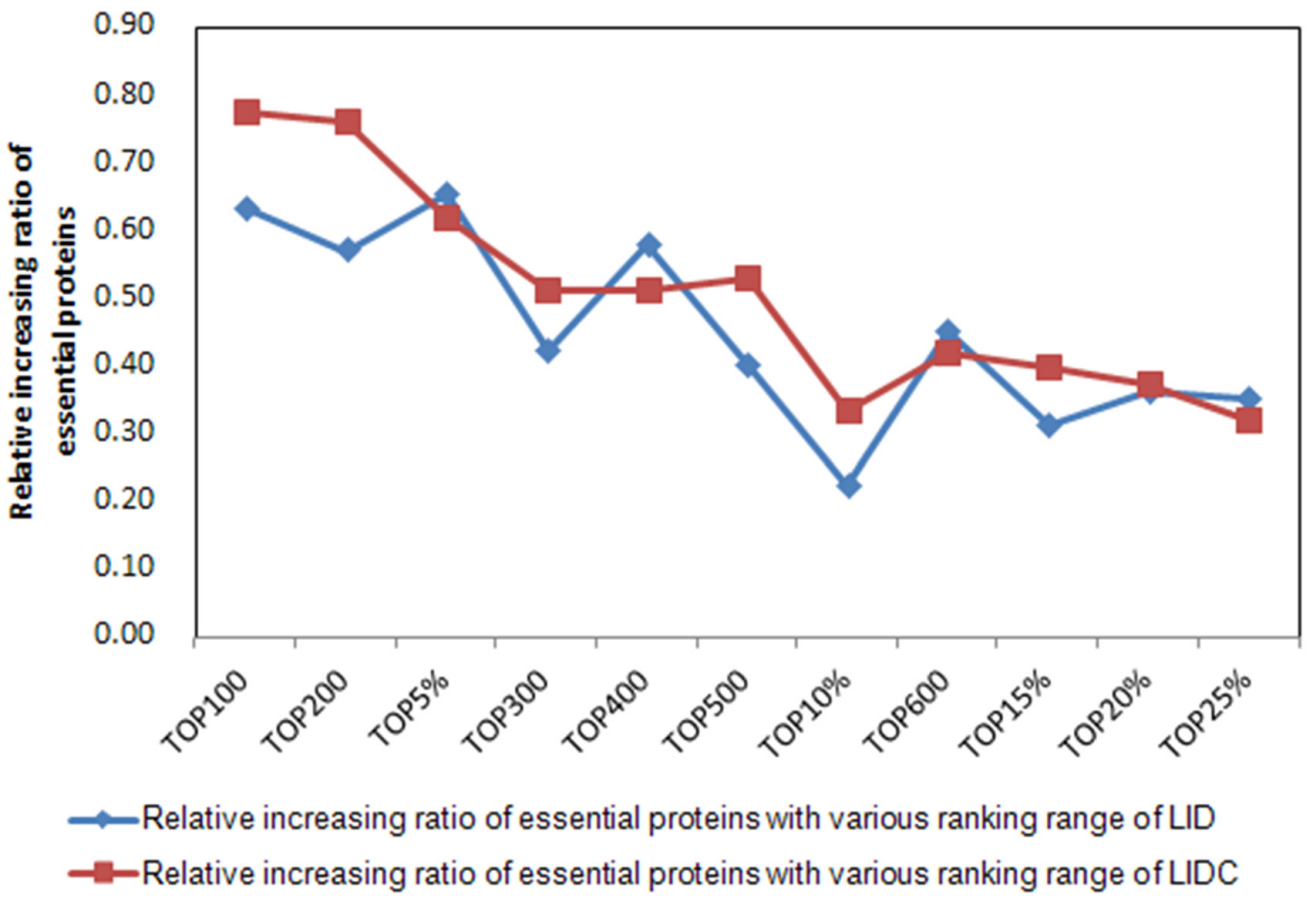
Comparison of the relative increasing ratio of essential proteins with various ranking ranges between LIDC and LID in the YDIP_5093 PIN. Relative increasing ratio = ΔLIDC/ΔTOP.

**Fig 9:**
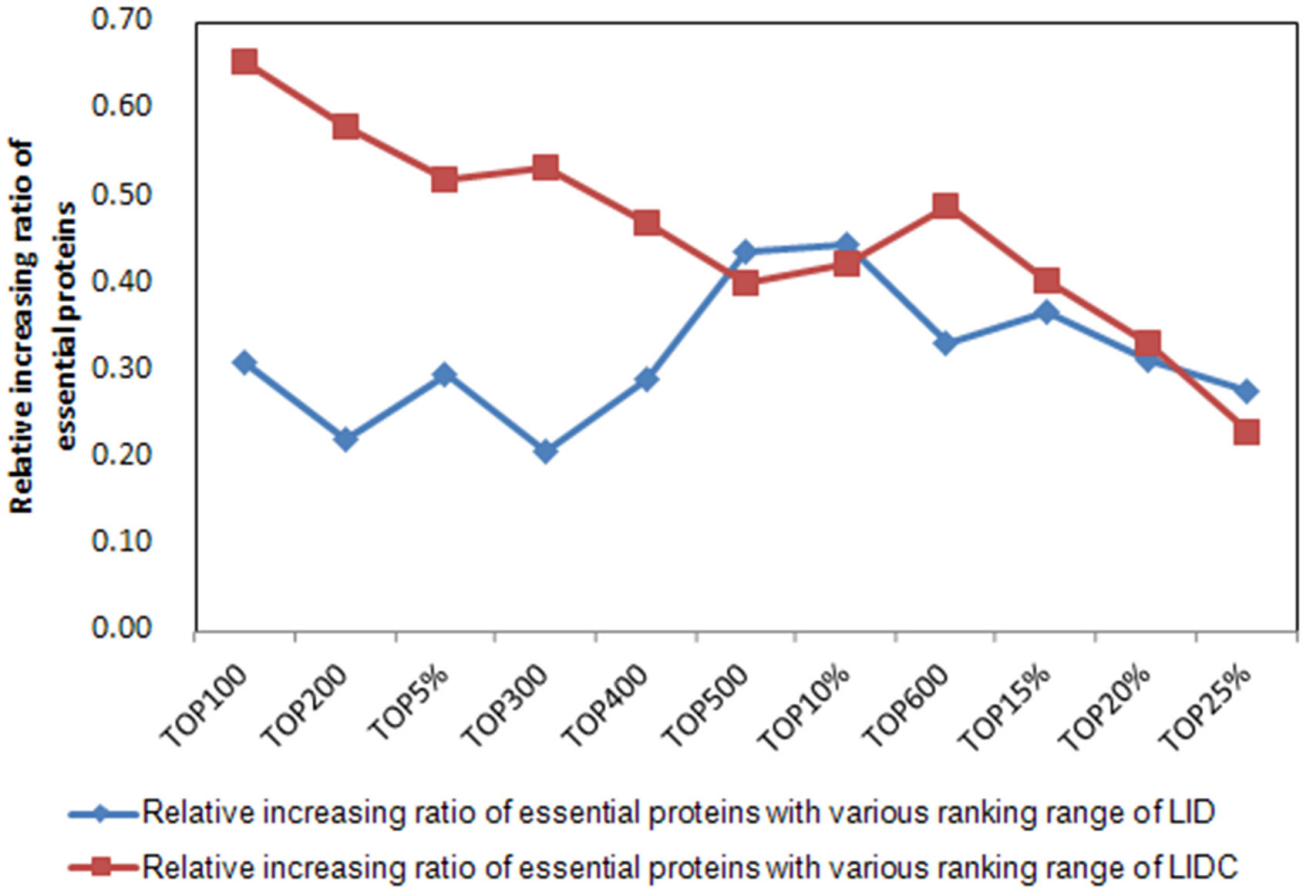
Comparison of the relative increasing ratio of essential proteins with various ranking ranges between LIDC and LID in the YMIPS_4546 PIN. Relative increasing ratio = ΔLIDC/ΔTOP.

From Fig 8, there were three relatively steady intervals in the 12 ranking ranges of YDIP_5093 for the relative increasing ratio curve of LIDC compared to that of LID, i.e., from top 100–200, top 300–500, and from top 600 to top 20%. The three intervals comprised eight ranking ranges, indicating that the performance of LIDC may improve steadily in 66.7% of all 12 ranges in YDIP_5093. The results for YMIPS_4546 were similar (Fig 9). There were also two relative steady intervals for the 12 ranking ranges (from top 5% to top 300 and top 500–600), comprising five ranking ranges to steadily improve the performance of LIDC. Thus, the new integration strategy of LIDC was suitable for decreasing the sensitivity of identification methods for network structures through improving the performance of LIDC in the middle intervals (i.e., from top 300–600) of ranking ranges with decreased impact of LID and increased impact of IDC on LIDC.

Above all, our new strategy may contribute to the enhancement of the LIDC identification method for proteins whose centrality values were located in the middle part of ranking ranges after descending sorting. The second advantage of this new strategy is that it decreases the number of unidentified proteins that have the same centrality scores, improving the performance of LIDC. For example, there were 2705 proteins whose NC centrality measures were 0 and 2734 proteins that had 0 score using the PeC multi-information fusion method on the same DIP dataset; in contrast, there were 2430 proteins with 0 values using our LIDC method, a 10% reduction in the number of unidentified proteins. The third advantage of this integration strategy is that LID(*u*) will be changed with different PINs, thereby changing RANK(*u*) and LIDC(*u*). This type of change may increase the flexibility of LIDC when analyzing different PINs.

### Analysis of the differences between LIDC and the nine other methods

In this section, we described some analyses of the differences between prediction results of LIDC and those of the nine other identification methods in order to elucidate the reasons for the improved performance of LIDC. We made a comparison from the top 100 predicted proteins of each method to analyze the relationships among LIDC and the nine other methods, and the number of differences in the top 100 proteins predicted by any two different methods is shown in Table 7. In Table 7, |M-LIDC| indicates the number of proteins identified by method M, not by LIDC, and {LIDC-M} indicates the set of proteins identified by LIDC, not by method M. The |M-LIDC| column shows that the differences between LIDC and the nine other methods were all more than 73% in YDIP_5093 and 61% in YMIPS_4546, indicating that LIDC was a different identification method than the nine other methods. Second, we assessed the proportion of essential proteins in the proteins identified by LIDC and other methods. The remaining columns in Table 7 show how many essential proteins were predicted out of all the different proteins identified by LIDC and other methods. The results showed that the percentage of essential proteins identified by LIDC was higher than that detected by other methods in both PINs consistently. In YDIP_5093, the percentages of essential proteins identified by LIDC for the eight different protein sets were all more than 71% except UC; in contrast, only PeC and ION obtained a percentage of more than 70%. In YMIPS_4546, the lowest percentage of essential proteins identified by LIDC for the nine different protein sets was 60%, while the average percentage obtained by the nine reference methods was less than 30%.

**Table 7:** Analysis of the differences between LIDC and the nine other methods for prediction of the top 100 proteins.

Data sets	Methods(M)	|M-LIDC|	Essential proteins in {M-LIDC}	Essential proteins in {LIDC-M}	Proportion of Essential proteins in {M-LIDC}	Proportion of Essential proteins in {LIDC-M}
**YDIP_5093**	**DC**	92	38	68	41%	**74%**
	**BC**	93	38	70	41%	**75%**
	**NC**	75	36	57	48%	**76%**
	**LID**	75	40	55	53%	**73%**
	**PeC**	77	56	56	73%	**73%**
	**CoEWC**	73	49	52	67%	**71%**
	**WDC**	73	46	53	63%	**73%**
	**ION**	79	58	56	73%	**71%**
	**UC**	44	30	30	68%	**68%**
**YMIPS_4546**	**DC**	83	12	55	14%	**66%**
	**BC**	83	12	54	14%	**65%**
	**NC**	63	8	49	13%	**78%**
	**LID**	61	10	51	16%	**84%**
	**PeC**	82	33	55	40%	**67%**
	**CoEWC**	74	30	50	41%	**68%**
	**WDC**	70	10	51	14%	**73%**
	**ION**	90	64	56	71%	**62%**
	**UC**	55	8	43	15%	**78%**

The results shown at the top of the table are for the YDIP_5093 PIN, while the results at the bottom of the table are for the YMIPS_4546 PIN.

Then, we explored the meanings of differences between LIDC and the nine other methods in more detail. There were 92 and 93 different proteins for DC and BC, respectively, compared to LIDC in the top 100 proteins of YDIP_5093. Additionally, the percentages of essential proteins for DC and BC were both 41%, while LIDC obtained 74% and 75% essential proteins. Moreover, in YMIPS_4546, DC and BC both obtained 83 different proteins compared to the top 100 proteins obtained by LIDC, with 14% essential proteins for both DC and BC, versus 64% and 65% for LIDC. Thus, the higher the DC or BC and the lower the LIDC at a single protein, the less likely the protein was to be essential.

According to these results, we consider that LIDC was more effective at predicting essential proteins at the network level.

### Assessment of the modularity of proteins predicted by LIDC and six other methods

**Fig 10:**
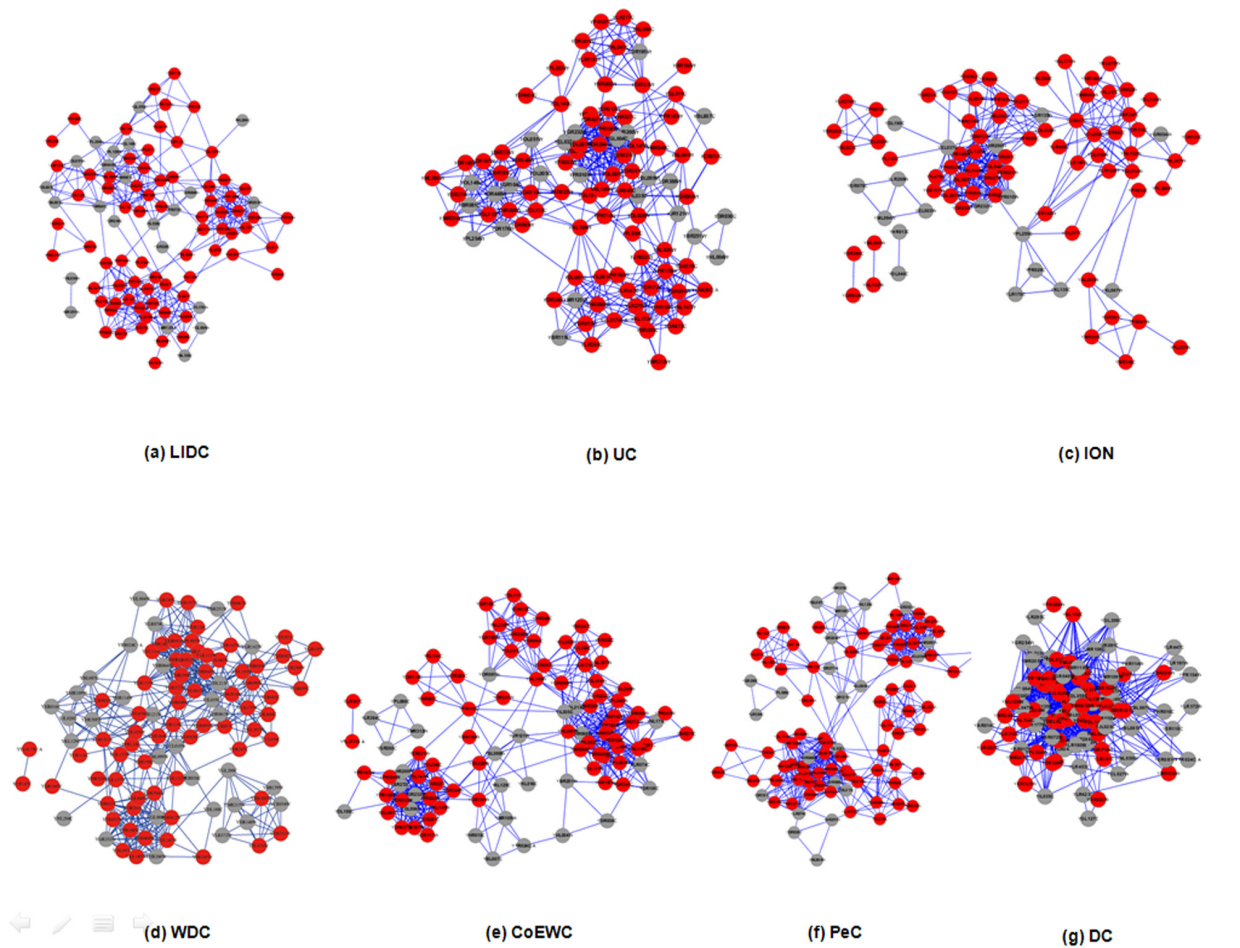
Seven subgraphs of the top 100 proteins without isolated nodes predicted through seven methods (LIDC, UC, ION, WDC, CoEWC, PeC, and DC) in the YDIP_5093 PIN, in which red nodes are essential proteins and grey nodes are nonessential proteins.

**Fig 11:**
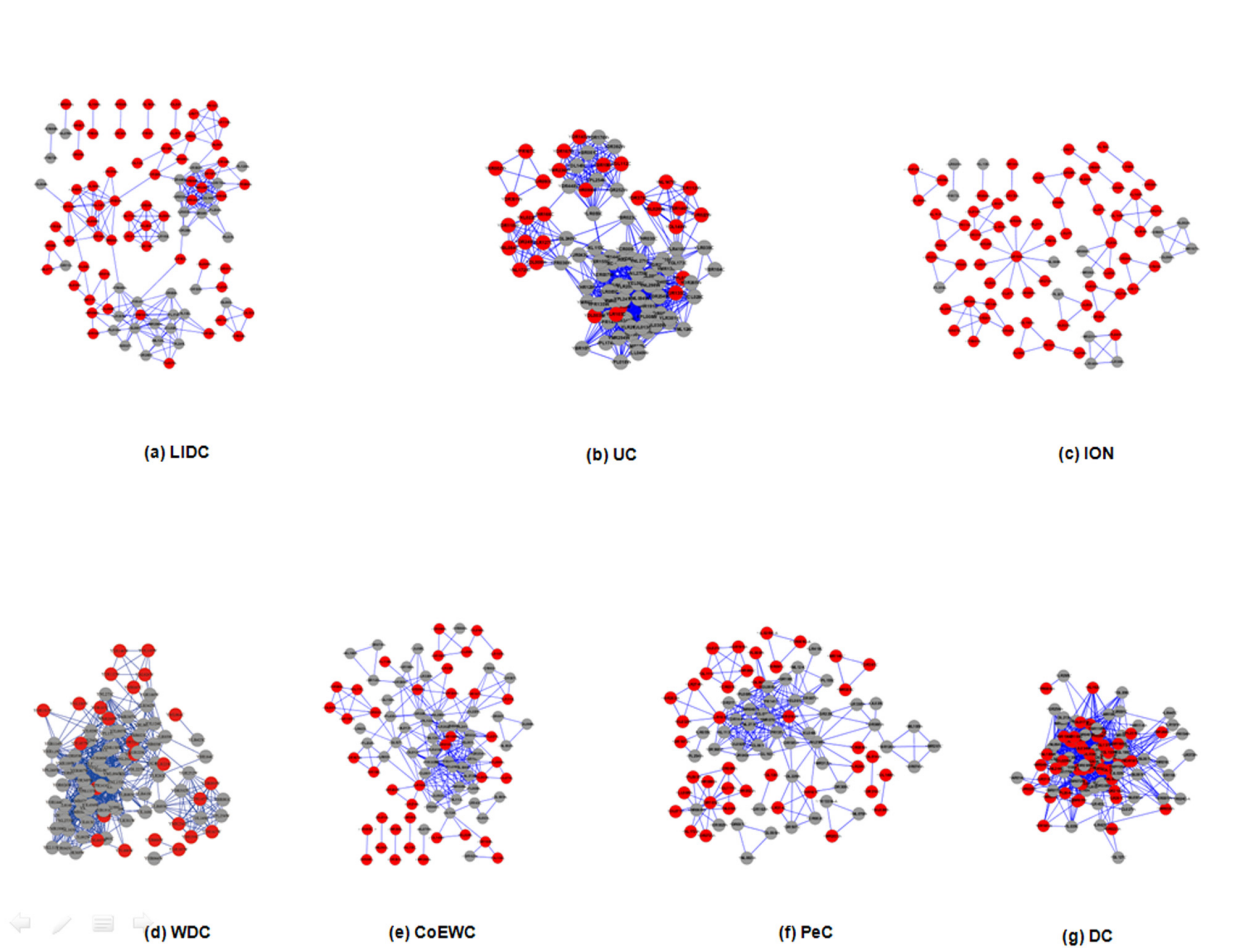
Seven subgraphs of the top 100 proteins without isolated nodes predicted through seven methods (LIDC, UC, ION, WDC, CoEWC, PeC, and DC) in the YMIPS_4546 PIN, in which red nodes are essential proteins and grey nodes are nonessential proteins.

Proteins rarely act alone; instead, proteins function in groups, i.e., protein complexes or functional modules, to perform their tasks in biological systems. Hence, protein modularity may be an appropriate approach to evaluate the rationality of essential proteins identified by LIDC in some biological sense. In order to explore the modularity of proteins predicted by LIDC and six other methods (i.e., UC, ION,WDC, CoEWC, PeC, and DC), whose probability to be essential were higher, each of seven small PINs were constructed based on the top 100 proteins detected by LIDC and the six reference methods in YDIP_5093 and YMIPS_4546. Each network consisted of the top 100 proteins ranked by LIDC and the six other methods. Next, we used clustering with the overlapping neighborhood expansion (ClusterONE) [42] to detect protein modules in these small PINs. In this analysis, we set a *p*-value of less than 0.001 in ClusterONE (*p*-value of less than 0.05 in the original ClusterONE paper) because we expected the protein modules predicted by ClusterONE to possess stronger biological significance, with the module size detected in more than two nodes. And seven top 100 network structures among LIDC and the six other methods are shown in Figs 10–11 respectively, which include LIDC,UC, ION, WDC, CoEWC, PeC, and DC.

As shown in Table 8, the number of edges (359 by LIDC) was the smaller in the seven top 100 PINs of YDIP_5093, and the average number of neighbors and network density, which were 7.18 and 5.14, respectively, were also smaller for the LIDC PIN. In contrast, the characteristic path length and number of essential proteins in the LIDC PIN were 3.30 and 76, both of which were the larger values for the seven top 100 PINs. These results indicated that the top 100 PIN of LIDC was a more sparse PIN than those of the six other methods and contained more essential proteins. Although more items of ION in Table 8 are smaller, both of the number of isolated nodes and essential proteins of ION are the largest in YDIP_5093 and YMIPS_4546. However the number of modules of ION is still smaller in two top 100 PINs respectively which has made the decrease of rationality of ION prediction.

**Table 8:** Statistical analyses of seven subgraphs of the top 100 proteins predicted by LIDC, DC, WDC, CoEWC, PeC, ION and UC.

Data sets	Methods	Number of Nodes	Number of Isolated Nodes	Number of Edges	NumberOf Essential Proteins	Characteristic Path Length	Average Number of Neighbors	Network Density	Number of Modules
**YDIP_5093**	**LIDC**	100	4	**359**	76	**3.30**	7.18	**0.07**	**7**
	**DC**	100	3	692	46	2.22	13.84	0.14	0
	**WDC**	100	0	484	69	2.98	9.68	0.1	4
	**CoEWC**	100	3	499	73	2.90	9.98	0.1	3
	**PeC**	100	3	496	76	3.09	9.92	0.1	5
	**ION**	100	14	306	78	3.19	6.12	0.06	2
	**UC**	100	1	513	76	2.92	10.26	0.1	4
**YMIPS_4546**	**LIDC**	100	4	**257**	66	**3.76**	5.14	**0.05**	**5**
	**DC**	100	1	671	23	2.36	13.42	0.14	3
	**WDC**	100	0	768	25	2.33	15.36	0.16	4
	**CoEWC**	100	8	266	46	3.06	5.37	0.06	2
	**PeC**	100	12	262	44	2.64	5.24	0.05	3
	**ION**	100	22	85	74	1.9	1.7	0.02	1
	**UC**	100	1	832	31	2.41	16.64	0.17	0

The results shown at the top of the table are for the YDIP_5093 PIN, while the results at the bottom of the table are for the YMIPS_4546 PIN.

Additionally, ClusterONE have detected seven functional modules in the PIN of LIDC, and LIDC yielded the best results in all seven top 100 PINs in YDIP_5093. We found that the more functional modules were in the more sparse top 100 PIN, which was identified by the LIDC method. Moreover, the same results were observed for the seven top 100 PINs of YMIPS_4546. Therefore, essential proteins predicted by LIDC possessed more obvious modularity than those identified by the six other methods in YDIP_5093 and YMIPS_4546 PINs. We still need to concern the effect of orthologous information of proteins for essential protein prediction according to performance of ION and it may be a reasonable way to combine LIDC and orthologous information based on our integrating strategy reported in this manuscript.

### Validated by protein interaction network of Escherichia coli

To further assess the performance of LIDC, we have taken used of it to predict essential proteins in *Escherichia coli* PIN. The PPI data of *E*. *coli* is downloaded from DIP database[33] called EDIP_2727. There are 2727 proteins and 11803 interactions whose self-interactions and repeated interactions have been deleted. The dataset of the essential proteins of *E*. *coli* has been collected from database DEG[36] which contains 296 essential proteins in which there are 254 proteins can be mapped in the PPI data of EDIP_2727. And then we consider the rest proteins in EDIP_2727 as nonessential proteins which has 2473 proteins.

Due to we have not obtained the real protein complex data of *Escherichia coli* PIN yet, we consider LIDC will be simplified to a pure LID measure to take part in comparative experiments with three reference measures which are PeC, CoEWC, WDC. The main reason for the choice of these three measures is the lack of necessary bioinformatics information for other rest reference multi information fusion methods.

The gene expression data of *Escherichia coli* has been downloaded from [43] which containing 7311 genes and 213 normalized affymetrix microarray gene expression profiles. There are 1970 proteins have the corresponding gene expression data while gene expression values of the rest 757 proteins of EDIP_2727 have been set to zero. The gene expression values of EDIP_2727 will participate in to compute PeC, CoEWC and WDC.

**Fig 12:**
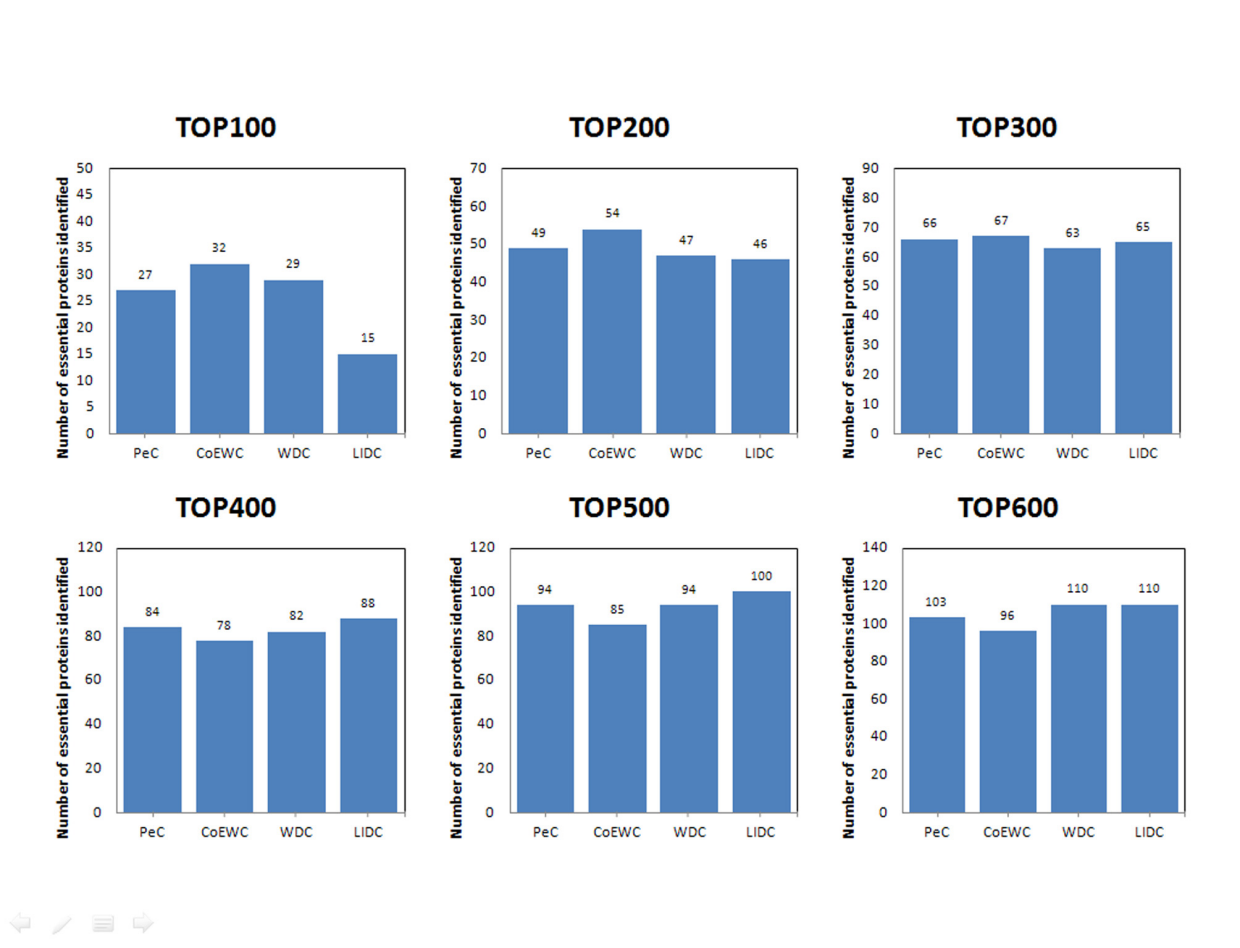
Comparison of the number of essential proteins from the top 100–600 identified by LIDC and three prediction measures in the EDIP_2727 PIN.

For evaluating the performance of LIDC in EDIP_2727 after ranking proteins in descending order based on their LIDC values and other methods, the six ranking ranges (from the top 100–600) were chosen as essential candidates. As shown in Fig 12, the performances of LIDC were better than three other methods for identifying essential proteins in three comparisons from top 400–600 although it is only a pure topological centrality in these comparisons, and this has confirmed the rationality of LIDC based on our analyses of the relationship between proteins and their neighbors in protein complexes and the partitions for nodes in protein interaction network.

For the sake of evaluate the overall performance of LIDC synthetically in EDIP_2727, we also have taken advantage of the same six statistical analysis parameters who include sensitivity, specificity, PPV, NPV, F-measure, and accuracy. The top 20% (545 proteins) selected from the ranking results of four measures were regarded as the essential protein candidate set, while the remaining results were considered the nonessential protein set (1282 proteins). The sensitivity, specificity, PPV, NPV, F-measure, and accuracy of LIDC were higher than those of the three other methods (Table 9). These data indicated that LIDC could identify essential proteins more accurately than the other tested methods in EDIP_2727 PIN.

**Table 9:** Comparison of the sensitivity (SN), specificity (SP), positive predictive value (PPV), negative predictive value (NPV), F-measure (F), and accuracy (ACC) between LIDC and the three other prediction methods for the top 20% of proteins in EDIP_2727 PIN.

Methods	SN	SP	PPV	NPV	F-measure	ACC
**PeC**	0.39	0.82	0.18	0.93	0.52	0.78
**CoEWC**	0.35	0.82	0.17	0.92	0.49	0.77
**WDC**	0.39	0.82	0.18	0.93	0.53	0.78
**LIDC**	**0.42**	**0.82**	**0.19**	**0.93**	**0.55**	**0.79**

To better understanding the performance of LIDC in protein interaction networks of *Escherichia coli*, AUC also has been consider a suitable measure to examine the power of LIDC on two-class classification in a PIN because it is an approximate imbalanced dataset of EDIP_2727 PIN where are 245 essential proteins and 1282 nonessential proteins. The AUC of LIDC (0.64) with respect to all proteins in EDIP_2727 PIN was the second best result among four prediction methods shown in Table 10. The performance of LIDC for this two-class classification for identification of essential proteins should be improved gradually with the accumulation of relative protein data.

**Table 10:** Comparisons of AUCs between LIDC and the three other methods in EDIP_2727 PIN.

Methods	EDIP_2727
**PeC**	0.52
**CoEWC**	0.55
**WDC**	0.65
**LIDC**	**0.64**

In order to assess the rationality of essential proteins identified by LIDC in some biological sense in *Escherichia coli*, we also has considered the protein modularity as an appropriate approach, and then explored the modularity of proteins predicted by LIDC and three other methods (i.e., LIDC, PeC, CoEWC, and WDC). Each of four small PINs were constructed based on the top 100 proteins detected by LIDC and the three reference methods in EDIP_2727 has been clustered with the overlapping neighborhood expansion (ClusterONE) [42] to detect protein modules in these small PINs. We still have set a *p*-value of less than 0.001 in ClusterONE, and the module size should be more than two nodes.

As shown in Table 11, the number of edges and the average number of neighbors of LIDC, which were 1389 and 27.78 respectively, were larger. And the characteristic path length and number of essential proteins in the LIDC PIN were 1.77 and 15, both of which were the smallest values in the four top 100 PINs. These results showed that the top 100 PIN of LIDC was a denser PIN than those of the three other methods so as to we expect to obtain more clusters. In contrast the number of clusters detected by ClusterONE is only four which is the smallest value in those of all four methods. Despite all that, the number of rational functional modules of LIDC in clusters were two which led to LIDC opposed the highest probability (2/4 = 0.5) for detecting rational functional modules in a top 100 PIN. Therefore, essential proteins predicted by LIDC possessed more modularity than those identified by the three other methods in EDIP_2727 PIN.

**Table 11:** Statistical analyses of four subgraphs of the top 100 proteins predicted by PeC, CoEWC, WDC, and LIDC in EDIP_2727 PIN.

Methods	Number of Nodes	Number of Isolated Nodes	Number of Edges	Number of Essential Proteins	Characteristic Path Length	Average Number of Neighbors	Number of Clusters	Number of Modules
**LIDC**	100	0	**1389**	15	**1.77**	27.78	**4**	**2**
**PeC**	100	1	644	27	2.45	12.9	16	1
**CoEWC**	100	3	763	32	2.23	15.26	14	2
**WDC**	100	0	1242	29	1.83	24.84	5	2

Although LIDC has a better performance in EDIP_2727 than three reference methods, there is no denying that ION is stronger than LIDC from the results in reference[15]. However it is a point we may concern that LIDC here is a just pure topological measure in virtue of the lack of protein complex data of *Escherichia coli*. Thus we still have confidences in the better improvement of LIDC in *Escherichia coli* with the accumulating of relative kinds of bioinformatics data gradually.

## Conclusions

In recent decades, there has been a sharp increase in PPI datasets due to the rapid development of experimental technologies, such as affinity purification. These experimental results may produce various PINs, and the identification of essential proteins at the network level has become a major focus of many researchers. Hence, many prediction methods have been proposed based on topological characteristics and various biological information of PINs. However, experiment datasets for many species are still incomplete, leading to false negative and false positive data. On the other hand, capturing the distinct features of essential proteins to improve the performance of identification is still challenging. These limits decrease the capabilities of existing prediction methods, making the prediction methods sensitive to the network structure of PINs. In order to overcome the constraints mentioned above, we explored real protein complexes to discover new characteristics of essential proteins with their neighbors in complexes, and then found that the essentiality of proteins may have a close relationship with interactions among proteins in the same clustering. As a result, we propose a new identification, LIDC, as a multi-information fusion method constructed by two information features, i.e., the LID and IDC of essential proteins, which represent new topological characteristics of proteins and in-degree information of protein complexes, respectively, based on the new integration strategy designed in this paper, with the goal of improving performance in multiple PINs.

LIDC was applied to three PINs of *S*. *cerevisiae* and *E*. *coli*: YDIP_5093, YMIPS_4546 and EDIP_2727. The experimental results indicated that LIDC outperformed recent classical identification methods, including DC, BC, NC, LID, PeC, CoEWC, WDC, ION and UC. In particular, we achieved better improvement in the number of essential proteins identified in YMIPS_4546. Additionally, experimental results also showed that LIDC was quite different from other existing methods and possessed higher accuracy. Moreover, the essential proteins identified by LIDC exhibited stronger modularity, such that the clusters connected by these proteins were also essential for special biological functions, consistent with recent studies [4, 18, 42, 44]. Hence, we concluded that LIDC could achieve better performance of identification methods synthetically through integration of multiple information datasets. In future studies, we will explore new suitable characteristics of essential proteins and examine how to combine multiple information datasets more effectively in order to increase the capability of LIDC.

## Supporting Information

S1 Excel Essential proteins and non-esential proteins data. (XLSX)

S2 Excel Protein complex data_745. (XLS)

S1 Text Protein interaction data in YDIP_5093. (TXT)

S2 Text Protein interaction data in YMIPS_4546. (TXT)

S3 Text Response of language editing track change. (DOC)
