# Supplementary material for: Identification of Essential Proteins Based on a New Combination of Local Interaction Density and Protein Complexes
Source: PLoS One. 2015 Jun 30;10(6):e0131418. doi: 10.1371/journal.pone.0131418 (PMC4488326; doi:10.1371/journal.pone.0131418)
Supplement: S3 Text — (DOC) [file pone.0131418.s005.doc]

Dear Editor,

Thank you for your useful comments and suggestions on the language of our manuscript. We have taken advantage of a professional scientific editing service with Editage ([www.editage.com](http://www.editage.com/)) for language problems in our manuscript which is one of partners of PLOS and our job code of Editage is YIQIZ_1, and then the language of our manuscript has been improved according to Editage’s advice. The copy of language editing track change of Editage is followed.

Identification of Essential Proteins Based on a New Combination of Local Interaction Density and Protein Complexes

Jiawei Luo^1,*^, Yi Qi^1^

^1^ College of Computer Science and Electronic Engineering, Hunan University, Changsha, China

*E-mail: luojiawei@hnu.edu.cn

**Abstract**

**Background**: Computational approaches aided by computer science have been used for prediction of proteins and are faster than expensive, time-consuming, laborious experimental approaches. However, the performance of such approaches is still poor, making practical applications of computational approaches difficult in some fields. Hence, the development of more suitable and efficient computing methods is necessary for identification of essential proteins.

**Method**: In this paper, we propose a new protein identification method, local interaction density combined with protein complexes (LIDC), based on statistical analyses of essential proteins and protein complexes. First, we introduce a new local topological property, local interaction density (LID), of the yeast PPI network; second, we discuss a new integration strategy for multiple bioinformatics. The LIDC method was then developed through a combination of LID and protein complex information based on our new integration strategy. The purpose of LIDC is discovery of important features of essential proteins in real protein complexes, thereby improving the efficiency of identification.

**Results**: Experimental results based on two different PPI networks (DIP and MIPS) of *Saccharomyces cerevisiae* showed that LIDC significantly outperformed classical topological centrality measures and some recent combinational methods. Moreover, when predicting MIPS datasets, more than 30% improvement was clearly obtained by LIDC over all seven reference methods (i.e., DC, BC, SoECC, LID, PeC, CoEWC, and WDC).

**Conclusions**: LIDC was more effective for the prediction of essential proteins than other recently developed methods.

**Introduction**

Proteins are vital parts of living organisms, functioning as the main components of the physiological metabolic pathways of cells, and form densely connected modules to perform various biological functions. With the development of proteomics research in the post-genomic era, several protein-related topics, including discovery of the structures and functions of proteins, detection of interactions among proteins, and identification of essential proteins or protein complexes and functional modules, have become the major focus of many researchers. Essential proteins are those proteins that, if deleted, result in lethality or infertility . Some recent results have suggested that comprehensive analyses of essential proteins will lead to a deeper understanding of the relationships between gene mutations and human diseases, thereby revealing the general principles of human diseases . Therefore, the prediction of essential proteins has important theoretical and practice significance in biological and medical fields.

Generally, two types of methods are used to identify essential proteins. The first is experimental techniques, such as single gene knockout , RNA interference , and conditional gene knockout , all of which are time-consuming and expensive. The second is bioinformatics computational approaches, which are faster and less expensive than experimental techniques, as supported by the sharp increase in the amount of high-throughput data available. Many computational approaches have been proposed for identification of the correlations among the topological properties of a protein, protein interaction networks (PINs), and the essentiality of proteins.

As a new modality for the identification of essential proteins, computational approaches focus on the topological properties of biological networks according to the centrality-lethality rule , and graph theory has become the main theoretical basis for construction of most computational approaches. There are about two subclasses in computational approaches: (1) topological centrality measures at the network level and (2) multi-information fusion measures, i.e., a combination of topological centrality measures and other biological information of proteins besides protein-protein interactions (PPIs), such as protein complexes , gene ontology (GO) terms of proteins , and gene expression data . However, the effectiveness of fusion strategies or mechanisms has not been sufficiently discussed. Therefore, it is critical to design suitable network-level methods integrated appropriately with biological information for prediction of essential proteins.

Existing results suggest that there is a correlation between essentiality and the degree of proteins in PINs, e.g., according to the centrality-lethality rule; this is also perhaps the main reason that most computational approaches must be sensitive to the topological structures of PINs. Additionally, subsequent experiments and analyses, e.g., yeast two-hybrid (Y2H) analyses, have shown that this type correlation may be too weak for binary or transient protein interactions . Moreover, Ryan et al. showed that entire complexes appear essential due to modular essentiality , and Wang et al. noted that larger protein complexes are more likely to be essential, explaining why essential genes are more likely to have high degrees of protein complex interactions . Genes in humans whose protein products belong to the same protein complex are more likely to be associated with the same disease phenotype . As shown in various studies, proteins tend to form densely connected modules with their adjacent nodes in order to perform specific biological functions, and essential proteins are the nodes in these modules that are required to maintain the existence of modules and biological functions . In this sense, in addition to topological properties of proteins, we may also pay attention to the correlation between the essentiality of proteins and protein complexes in PINs.

To overcome the limitations of existing computational approaches in current research on prediction of essential proteins, we have explored the interaction relationships among nodes in real protein complexes for the first time and subsequently proposed a new identification method, local interaction density combined with protein complexes (LIDC), which is composed of three components. The first is a new centrality measure based on the local topological properties of proteins in protein complexes, called the local interaction density (LID); the second is the biological information of protein complexes, called the in-degree centrality of complex (IDC); and the third is a new integration strategy for combining LID and IDC. LID is expected to facilitate the identification of essential proteins from their neighbors, which interact with other neighbors. LIDC was applied to two PIN datasets of *Saccharomyces cerevisiae*, a representative model organism, downloaded from the DIP and MIPS databases.

Compared to seven conventional methods, i.e., degree centrality (DC) , betweenness centrality (BC) , sum of edge clustering coefficient (SoECC) , local interaction density (LID), PeC , co-expression weighted by clustering coefficient (CoEWC) , and weighted degree centrality (WDC) , of which DC, BC, and SoECC are well-known topological centrality measures and PeC, CoEWC, and WDC are recent multi-information fusion measures that have shown good performance for protein identification, LIDC exhibited better performance for the identification of essential proteins based on comprehensive analyses of sensitivity, specificity, F-measures, positive predictive value, negative predictive value, accuracy, and area under receiver operating characteristic (ROC) curve (AUC). In particular, when predicting MIPS datasets, more than 30% improvement was clearly obtained by LIDC over all seven reference methods for every parameter measured.

**Materials and Methods**

**Motivation**

Most existing prediction methods are built on the centrality-lethality rule and the topological centrality of PINs; even recent multi-information fusion measures, which often perform better than purely topological centrality methods, usually include one type of topological centrality measure. To further improve the prediction capability of multi-information fusion measures, we may be confronted with two problems: 1) discovery of new topological centralities, and 2) development of more effective integration strategies.

With regard to existing topological centrality, we have learned that most high-degree hub nodes are not essential in yeast PINs . The correlation between essentiality and topological centrality of single proteins in PINs may be weak for binary or transient protein interactions . On the other hand, proteins tend to form protein complexes with their adjacent nodes in order to perform specific biological functions. Hence, in this study, we explored, for the first time, the relationship between properties of proteins in real complexes and essentiality of proteins, rather than just considering the topological centrality of proteins at the global network level.

Through statistical analysis of the identification methods mentioned above, we noticed that the performance of topological centrality measures was better within the top 100–600 rankings, while that of multi-information fusion measures was best for the top 1%–25% of rankings. Moreover, many proteins have the same centrality score in a given ranking range predicted by one method, and these proteins may not be distinguished clearly, regardless of whether the protein is essential or nonessential. These limits may reduce the overall performance of existing identification methods.

In order to achieve improved performance of identification methods, we present herein a new integration strategy that combines LID and information of protein complexes in order to construct a new method, termed LIDC, which achieved outstanding performance in 12 ranking ranges: top 100, top 200, top 300, top 400, top 500, top 600, top 1%, top 5%, top 10%, top 15%, top 20%, and top 25%.

**Definition of the problem and analyses**

A PIN is generally described as an undirected graph $G=(V,E)$, which consists of a node set *V* and an edge set *E*. One node $v\in V$ represents a unique protein, while one edge $(u,v)\in E$ represents an interaction between protein *u* and protein *v*. The adjacency matrix is written as *A* of *G*, and the matrix (an *N × N* symmetric matrix) is written as$A=a_{u,v}$, where the variable N is the total number of nodes in a PIN; thus,

$$a_{u,v}=\left\{\begin{matrix} 1, (u,v)\in E\text{;} \\ 0, \text{otherwise.} \end{matrix}\right.$$

$G_{\text{NB}}(u)=(V_{\text{NB}}(u),E_{\text{NB}}(u))$ is the adjacency subgraph of a protein *u*, i.e., a source node of $G_{\text{NB}}(u)$ in a PIN, where $V_{\text{NB}}(u)$is the node set in $G_{\text{NB}}(u)$, and $E_{\text{NB}}(u)$ is the edge set in $G_{\text{NB}}(u)$. $V_{\text{NB}}(u)$ is the union of two neighbor node subsets, $V_{\text{NB\_ISO}}(u)$ and $V_{\text{NB\_INT}}(u)$, while $E_{\text{NB}}(u)$ is also the union of two neighbor edge subsets, $E_{\text{NB\_ADE}}(u)$ and $E_{\text{NB\_INT}}(u)$. Edges in $E_{\text{NB\_ADE}}(u)$ are called adjacency edges and are interactions between a source node *u* and all of its neighbors; edges in $E_{\text{NB\_INT}}(u)$ are called interactive edges and are interactions among neighbor nodes of *u*. Nodes in $V_{\text{NB\_ISO}}(u)$, called isolated neighbors, are nodes that only have adjacency edges to the source node *u*, while nodes in $V_{\text{NB\_INT}}(u)$, called interactive neighbors, are nodes that have both adjacent edges to *u* and interactive edges, as shown in Fig. 1. A node *u* is a star node if its $V_{\text{NB\_INT}}(u)$ is empty and the topological structure of its adjacent subgraph is a star, e.g., protein YDL171C, as shown in Fig. 2.

**Fig. 1. Protein YJL143W (red) is a no-star node with its five neighbors.** VNB(u) = VNB_ISO(u)∪VNB_INT(u), u = YJL143W, VNB_INT(u) = {YNR017W,YJR045C, YNL131W,YMR203W}, VNB_ISO(u) = {YJL064W}. Five blue long dashes represent the adjacent edges between YJL143W and its neighbors, and the two black solid lines represent interactive edges among interactive neighbors colored by purple. Green nodes are isolated neighbors, such as protein YJL064W. Protein YJL143W is an essential protein.

**Fig. 2. Protein YDL171C (red) is a star node with its five neighbors.** The blue long dashes are the adjacent edges between the source node YDL171C and its green neighbors, which are completely isolated, meaning they will be isolated points in the adjacency subgraph of u if these adjacent edges can be ignored. Protein YDL171C is a nonessential protein.

In order to analyze the correlations between interactive neighbors and essential proteins in terms of modularity, we first examined nodes in a real protein complex dataset based on our partition of nodes. In a yeast protein complex dataset containing 408 complexes and 1920 nodes, without considering the overlap among complexes (whose interaction information was obtained from a yeast protein interaction network downloaded from the DIP database, which has 5616 proteins and 52833 interactions ), there were 140 essential proteins in 435 isolated neighbors whose in-degree in the complex was 1 (7% of 1920 nodes). Moreover, there were 1485 nodes which were interactive neighbors in complexes, with in-degrees of more than 1; 681 of these nodes represented essential proteins (35% of 1920 nodes). Additionally, according to degree analysis of nodes in the yeast PIN mentioned above, there were 2705 star nodes, of which only 13.1% were essential proteins, and 2388 non-star nodes (indicating the presence of some interactive neighbors), out of which 34% were essential proteins, e.g., protein YJL143W in Fig. 1. Therefore, we proposed a new local topological measure, LID, based on protein complexes, to determine the essentiality of a protein by evaluating the relationship between a protein and its interactive neighbors.

**New identification algorithm: LIDC**

LIDC is a multi-information fusion measure proposed in this paper that is composed of three parts as follows.

**1) A new topological centrality: LID centrality**

The LID of a node *u* (LID(*u*)) is defined as the density of interactions among its interactive neighbors:

$\text{LID}(u)=\frac{\left|E_{\text{NB\_INT}}(u)\right|}{\left|V_{\text{NB\_INT}}(u)\right|}$ (1)

where the operator || is a count of the number of elements in a set. For example, in Fig. 1, node *u* is protein YJL143W, and we then have $\left|E_{\text{NB\_INT}}(u)\right|=2$ and $\left|V_{\text{NB\_INT}}(u)\right|=4$, such that $\text{LID}(u)=0.5$. Obviously, LID(*u*) will be larger if there are fewer neighbors of node *u*, resulting in denser interactions among the nodes.

**2) IDC**

Protein complexes are stable macromolecular assemblies that perform many diverse biochemical activities essential to cell homeostasis, growth, and proliferation; proteins within these complexes appear at the same time and in the same location, and more some proteins participate in more than one complex. At the network level, protein complexes are usually substructures in PINs. In our design, we may compute the IDC of a protein to identify the essential proteins. The IDC of a protein *u* in a protein complex set is defined as:

$\text{IDC}(u)=\sum_{i\in ComplexSet(u)} \text{IN-Degree}(u)_{i}$ (2)

where ComplexSet(*u*) denotes a set of protein complexes that include protein *u*, and IN-Degree(*u*)*_i_* is the in-degree value of protein *u* in *i*th protein complex that belongs to ComplexSet(*u*). IN-Degree(*u*)*_i_* is computed as:

$\text{IN-Degree}(u)_{i}=DC(u)_{i}$ (3)

where DC(*u*)*_i_* is the degree value of protein *u* in complex *i*, which is *i*th element in ComplexSet(*u*).

**3) Integration strategy of LID and IDC**

The purpose of our new integration strategy is to overcome the constraints of existing identification methods as follows. First, the performance of existing identification methods is not consistently good for 12 ranking ranges (from top 100 to top 25%); in general, topological centrality measures are good from the top 100–600, while multi-information fusion measures are good from the top 1%–25%. Second, existing identification methods lack sufficient flexibility to adapt to various network structures of PINs. The construction of topological centrality measures is based on a special dataset, generally becoming the innate deficiency in multiple datasets, indicating that the performance of this type of identification method is sensitive to the structures of PINs. There are two types of integration strategies used to combine multi-information fusion measures. The first is that the prediction score of proteins is the product of values of multifeature information, such as PeC and CoEWC, and the second is that the predicting score of proteins is the linear sum of values with some coefficients of multifeature information, such as WDC. The way of product increases the network structure dependence of those methods than the linear sum way without sufficient guarantee of the quality of the dataset, leading to high false positives and false negatives of interactions among proteins. On the other hand, the pattern of the linear sum contains some coefficients that adjust the proportions of feature values for each type of information in the predicting score of the proteins, and however many of these coefficients are constants, which, in most cases, depend on a given PIN, thereby increasing the network structure sensitivities of these methods as well. Therefore, we consider a new integration strategy to pursue good performance in all 12 ranking ranges at the same time in order to improve flexibility in the context of multiple PINs.

The new integration strategy we consider here has the advantages of the topological centrality measure LID and the prediction method IDC. Let RANK(*u*) be the order number of the descending sort of protein *u* according to LID(*u*) in a PIN. When LID(*u*) is larger and RANK(*u*) is smaller, ~~which is the order number of the descending sort of protein~~ *~~u~~* ~~according to LID(~~*~~u~~*~~) in a given PIN,~~ LIDC(*u*) mainly depends on LID values. However, when LID(*u*) is smaller and RANK(*u*) is larger, LIDC(*u*) depends on IDC values, suggesting that the local topological properties of the current protein are useless for prediction and that more biological information, such as IDC, is required.

**LIDC algorithm for identification of essential proteins**

After summarizing and analyzing the experimental results of most topological centrality measures, including our new centrality LID, we found that there was no single centrality measure that could predict all essential proteins correctly and completely in a given PIN. Therefore, we must construct multi-information fusion measures in order to improve the performance of identification synthetically. Thus, we chose the IDC as our second biological information source in our new identification method because of the modularity of protein function, as mentioned above. Therefore, the LIDC of protein *u* can be computed as:

$\text{LIDC}(u)=LID(u)\times(1-\frac{RANK(u)}{N})+IDC(u)\times\frac{RANK(u)}{N}$ (4)

where LID(*u*) is the value of the LID, IDC(*u*) is the value of IDC of the protein complex of protein *u*, and *N* is the number of proteins in the current PIN. RANK(*u*) is the order number of the descending sort of protein *u* according to LID(*u*) in the current PIN, which can vary with different datasets such that LIDC can be more flexible and adjustable than traditional, simple, empirical, single-parameter patterns.

From the definition and analyses of LIDC above, LIDC can identify essential proteins more effectively than existing identification methods.

**Results and Discussion**

**Experimental dataset**

To evaluate the performance of our proposed LIDC method, we considered PPI data of *S. cerevisiae* (yeast) as experimental material because this model organism has complete, reliable PPI and essential protein data. The PPI network of *S. cerevisiae* was downloaded from two databases: the DIP database , shown in S1 File, and the MIPS database , shown in S2 File. A collection of essential proteins of *S. cerevisiae* was gathered from several databases, including MIPS , SGD , DEG , and SGDP , yielding 1285 essential proteins and 4394 nonessential proteins (S3 File).

In this study, we integrated four real protein complex sets (CM270, CM425, CY408, and CY428) into one comprehensive protein complex set. Of the four protein complexes datasets, only complexes with a size of two or more were kept. CM270 is the gold standard protein complex set and was downloaded from the MIPS database . It contains 270 complexes and 1230 proteins. The second real complex set (CM425), described by Friedel et al , was integrated from three sources, i.e., MIPS , Aloy et al , and the SGD database , and contains 425 complexes and 1970 proteins. The third and fourth complex sets (CYC408 and CYC428) were obtained from CYC2008 of the Wodak Laboratory . We combined these four protein complex datasets to obtain a new complex dataset containing 745 protein complexes (comprised of 2167 proteins); we called this dataset Complex_745 (S4 File). Note that data for these protein complexes were detected through experimental methods and thus did not contain all proteins within the given PPI networks. This is an inherent defect of IDC that limits the use of IDC as a stand-alone independent identification method.

Next, we constructed two yeast PINs for evaluating LIDC from two PPI datasets whose nodes represented proteins and edges represented interactions among proteins. The first network downloaded from the DIP database, marked YDIP_5093, included 5093 proteins and 24743 interactions. Its average degree was about 9.72. The second network downloaded from the MIPS database, marked YMIPS_4546, included 4546 proteins and 12319 interactions. Its average degree was about 5.42. The numbers of interactions in the two PINs were counted after removing self-interactions and duplicate interactions. With regard to the essentiality of proteins in a PIN, nodes were grouped into three subsets in a network: essential proteins, nonessential proteins, and unknown proteins covered by neither the essential protein set nor the nonessential protein set. We incorporated the unknown protein set into the nonessential protein set for the sake of easier discussion in this paper. There were 1167 essential proteins and 3926 nonessential proteins in network YDIP_5093 and 1016 essential proteins and 3530 nonessential proteins in network YMIPS_4546. The proportion of essential proteins in each PIN was nearly equal, which helped us to examine the precision of LIDC rationally. The details of nodes within the two PINs are shown in Table 1.

The global characteristics of the two PINs were similar for most items in Table 2, except for the average degree, which was 9.72 in YDIP_5093 and 5.42 in YMIPS_4546 (i.e., 1.79 times higher in YDIP_5093 than in YMIPS_4546), suggesting that YMIPS_4546 may be more sparse and more difficult to use for prediction than YDIP_5093.

From local topological characteristics of PINs shown in Table 3, we found that average values of interactive edges, interactive neighbors, and isolated neighbors in YDIP_5093 were roughly two times higher than those in YMIPS_4546, while the heterogeneities of interactive edges, interactive neighbors, and isolated neighbors in YMIPS_4546 were 2–4 times higher than those in YDIP_5093. The heterogeneity of interactive edges was 42.64 in YMIPS_4546, indicating that the distribution of the interactive edges of nodes was quite unbalanced in this PIN. These heterogeneities of interactive edges suggested that it was not easy for LIDC to achieve better performance in YMIPS_4546.

| **Table 1. The two yeast protein-protein interaction networks obtained from two PPI datasets (DIP and MIPS).** | | | | | | |
| --- | --- | --- | --- | --- | --- | --- |
| **Data sets** | **Data sources** | **Proteins** | **Interactions** | **Essential proteins** | **Non-essential**  **proteins** | **Unknown**  **proteins** |
| **YDIP_5093** | DIP | 5093 | 24743 | 1167 | 3591 | 335 |
| **YMIPS_4546** | MIPS | 4546 | 12319 | 1016 | 3195 | 335 |
|  |  |  |  |  |  |  |

| **Table 2. The global characteristics of two yeast protein interaction networks.** | | | | | |
| --- | --- | --- | --- | --- | --- |
| **Data sets** | **Max degree** | **Average degree** | **Heterogeneity of degree** | **Nodes in max connected component** | **Average distance in max connected component** |
| **YDIP_5093** | 280 | 9.72 | 4.01 | 5052 | 3.84 |
| **YMIPS_4546** | 286 | 5.42 | 5.91 | 4385 | 4.42 |
| *Heterogeneity(v)=average(v^2^)/(average(v))^2^* | | | | | |
|  | | | | | |

| **Table 3. The local characteristics of two yeast protein interaction networks.** | | | | | | |
| --- | --- | --- | --- | --- | --- | --- |
| **Data sets** | **Average  interactive edges** | **Average  interactive neighbors** | **Average  isolated neighbors** | **Heterogeneity of interactive edges** | **Heterogeneity of interactive neighbors** | **Heterogeneity of isolated neighbors** |
| **YDIP_5093** | 10.75 | 5.45 | 4.27 | 10.52 | 5.85 | 3.77 |
| **YMIPS_4546** | 6.76 | 2.48 | 2.93 | 42.64 | 12.28 | 6.18 |
|  |  |  |  |  |  |  |
|  |  |  |  |  |  |  |
|  |  |  |  |  |  |  |

**Evaluation methods**

In general, topological centrality measures may obtain good results for the top 100–600 rankings, while multi-information fusion methods usually have better performance for the top 1%–25% of rankings. The reason that top 25% was a maximum of the ranking range was that the top 25% of YDIP_5093 and YMIPS_4546, i.e., 1273 and 1136 proteins, respectively, was close to the number of essential proteins in the collection of essential proteins of *S. cerevisiae* mentioned above (i.e., 1285) and was beneficial for predicting performance.

To evaluate the overall performance of LIDC, we first ranked the results of LIDC in descending order and then chose proteins in 12 different ranking ranges (from top 100 to top 25%) as potential essential protein sets of the two PINs. The more essential proteins were identified in each potential set for the given method, the better the performance of the method.

For assessing the effectiveness and accuracy of LIDC, we selected the top 20% of ranking results from LIDC, which was regarded as the essential protein candidate set, and the last of the results, which were considered nonessential protein sets, because the number of proteins in the top 20% of the two PINs was relative equal. We then calculated several parameters, including the values of true positives (TPs), true negatives (TNs), false positives (FPs), false negatives (FNs), and obtained the outcomes of six common statistical methods using these values (TP, TN, FP, and FN), which are usually used to evaluate the effectiveness of an algorithm. Sensitivity was defined as $SN=TP/(TP+FN)$; specificity was defined as $SP=TN/(TN+FP)$; positive predictive value (PPV) was defined as $PPV=TP/(TP+FP)$; negative predictive value (NPV) was defined as $NPV=TN/(TN+FN)$; F-measure was defined as $F=2\times SN\times PPV/(SN+PPV)$; accuracy was the proportion of correct prediction results of all the results, which is defined as $ACC=(TP+TN)/(Posi+Nega)$, where *Posi* and *Nega* are the total number of essential proteins and nonessential proteins, respectively. In addition, identification of essential proteins was also considered a classification problem; therefore, the area under the receiver operating characteristic (ROC) curve (AUC) was a suitable measure used to estimate the performance of our new method. The expression of AUC was calculated as :

$AUC=\frac{\sum_{u\in\text{Essential} \text{Protein}} rank(u)-\frac{1}{2}Posi(Posi+1) }{Posi\times Nega}$ (5)

where *rank(u)* is the descending order of the essential proteins *u*. Then, we compared LIDC with the other seven existing prediction measures to determine which had the best performance for the two PINs.

**Comparison of LIDC with other centrality measures**

In order to validate the performance of LIDC, a comparison was carried out with seven classical identification measures: DC, BC, SoECC, PeC, CoEWC, and WDC. Among these, DC and BC are classic local and global topological centrality measures, respectively, and have been used by many researchers in various fields . Additionally, SoECC is a core measure used for some multi-information fusion measures and a good local topological centrality measure . PeC was recently reported in 2012 and is based on the integration of PPI data and gene expression data. Moreover, PeC outperforms 15 other centrality measures in the yeast PPI network , including DC. CoEWC and WDC are two of the most recently reported multi-information fusion measures; CoEWC, proposed in 2013, may capture the properties of both data hubs and party hubs despite that the two hubs have very different clustering properties. WDC, proposed in 2014, is used to show the linear correlation of topological centrality and relevant gene expression data.

For the purpose of performance testing of LIDC after ranking proteins in descending order based on their LIDC values and other methods, the 12 ranking ranges (from the top 100 to the top 25%) were chosen as essential candidates, and finally, the essentiality of the proteins was checked through the collection of essential proteins described in the *Experimental dataset* section. The numbers of essential proteins predicted by LIDC and the other seven methods in the two yeast networks are shown in Figs. 3–6; Figs. 3 and 4 report the results for the top 100–600 and the top 1%–25%, respectively, in YDIP_5093, while Figs. 5 and 6 report the results in YMIPS_4546.

As shown in Figs. 3 and 4, the performance of LIDC were better than all seven other methods for identifying essential proteins in 11 of 12 comparisons from the top 100 to the top 25% in dataset YDIP_5093; the exception was the top 1%, for which LIDC ranked third in performance. Moreover, we observed an average 3% improvement for predicting essential proteins by LIDC compared with the best results of the other seven methods in the 12 comparisons (Table 4).

As shown in Figs. 5 and 6, the performance of LIDC was better than all seven other methods for identifying essential proteins for all 12 comparisons from the top 100 to the top 25% ranges in dataset YMIPS_4546. In particular, LIDC yielded more than 40% improvement over the best results of the seven other methods for predicting the top 300 and top 400. Moreover, there was an average 37% improvement for predicting essential proteins by LIDC over the best results of the seven other methods in the 12 comparisons (Table 4). Thus, experiments indicated that LIDC could identify more essential proteins than reference methods.

**Fig. 3. Comparison of the number of essential proteins from the top 100–600 identified by LIDC and seven other prediction measures in the YDIP_5093 PIN.**

**Fig. 4. Comparison of the number of essential proteins from the top 1%–25% identified by LIDC and seven other prediction measures in the YDIP_5093 PIN.**

**Fig. 5. Comparison of the number of essential proteins from the top 100–600 identified by LIDC and seven other prediction measures in the YMIPS_4546 PIN.**

**Fig. 6. Comparison of the number of essential proteins from the top 1%–25% identified by LIDC and seven other prediction measures in the YMIPS_4546 PIN.**

| **Table 4. Statistical analyses of improvement according to comparisons between**  **LIDC and the best results of the seven other methods in each ranking range.** The  results at the top of the table are for the YDIP_5093 PIN, and the rest are for the YMIPS  _4546 PIN. | | | | | |
| --- | --- | --- | --- | --- | --- |
| **Ranking range** | **Best result of**  **the other**  **seven methods** | **LIDC** | **Values of Improvement** | **Proportion of Improvement** | **Average proportion of Improvement** |
| **TOP100** | 76 | **76** | 0 | 0% | **3%** |
| **TOP200** | 144 | **152** | 8 | 6% |  |
| **TOP300** | 203 | **209** | 6 | 3% |  |
| **TOP400** | 255 | **260** | 5 | 2% |  |
| **TOP500** | 300 | **313** | 13 | 4% |  |
| **TOP600** | 341 | **354** | 13 | 4% |  |
| **TOP1%** | 41 | **38** | -3 | -7% |  |
| **TOP5%** | 177 | **186** | 9 | 5% |  |
| **TOP10%** | 306 | **316** | 10 | 3% |  |
| **TOP15%** | 401 | **411** | 10 | 2% |  |
| **TOP20%** | 487 | **514** | 27 | 6% |  |
| **TOP25%** | 574 | **595** | 21 | 4% |  |
| **TOP100** | 46 | **66** | 20 | 43% | **37%** |
| **TOP200** | 91 | **124** | 33 | 36% |  |
| **TOP300** | 123 | **177** | 54 | 44% |  |
| **TOP400** | 160 | **224** | 64 | 40% |  |
| **TOP500** | 195 | **265** | 70 | 36% |  |
| **TOP600** | 230 | **314** | 84 | 37% |  |
| **TOP1%** | 22 | **30** | 8 | 36% |  |
| **TOP5%** | 102 | **138** | 36 | 35% |  |
| **TOP10%** | 180 | **246** | 66 | 37% |  |
| **TOP15%** | 259 | **347** | 88 | 34% |  |
| **TOP20%** | 312 | **422** | 110 | 35% |  |
| **TOP25%** | 369 | **474** | 105 | 28% |  |
|  |  |  |  |  |  |

**Verification of six statistical methods**

In order to evaluate the overall performance of LIDC synthetically, we took advantage of six statistical analysis parameters, including sensitivity, specificity, PPV, NPV, F-measure, and accuracy. The top 20% selected from the ranking results of all methods was regarded as the essential protein candidate set, while the remaining results were considered the nonessential protein set (1019 and 909 proteins, respectively, for the two PINs). The sensitivity, specificity, PPV, NPV, F-measure, and accuracy of LIDC were consistently higher than those of the seven other methods for both PINs (Table 5). These data indicated that LIDC could identify essential proteins more accurately than the other tested methods.

**Validation by the AUC**

| **Table 5. Comparison of the sensitivity (SN), specificity (SP), positive predictive value (PPV), negative predictive value (NPV), F-measure (F), and accuracy (ACC) between LIDC and the seven other prediction methods for the top 20% of proteins in two different PINs.** | | | | | | | |
| --- | --- | --- | --- | --- | --- | --- | --- |
|  |  |  |  |  |  |  |  |
|  |  |  |  |  |  |  |  |
| **Data sets** | **Methods** | **SN** | **SP** | **PPV** | **NPV** | **F-measure** | **ACC** |
|  | DC | 0.35 | 0.85 | 0.41 | 0.81 | 0.50 | 0.73 |
|  | BC | 0.31 | 0.83 | 0.35 | 0.80 | 0.45 | 0.71 |
|  | SoECC | 0.40 | 0.86 | 0.46 | 0.83 | 0.54 | 0.75 |
| **YDIP_5093** | LID | 0.39 | 0.86 | 0.45 | 0.83 | 0.54 | 0.75 |
|  | PeC | 0.40 | 0.86 | 0.46 | 0.83 | 0.55 | 0.75 |
|  | CoEWC | 0.41 | 0.86 | 0.47 | 0.83 | 0.56 | 0.76 |
|  | WDC | 0.42 | 0.86 | 0.48 | 0.83 | 0.56 | 0.76 |
|  | LIDC | **0.44** | **0.87** | **0.50** | **0.84** | **0.59** | **0.77** |
|  | DC | 0.25 | 0.82 | 0.28 | 0.79 | 0.38 | 0.69 |
|  | BC | 0.25 | 0.81 | 0.28 | 0.79 | 0.38 | 0.69 |
|  | SoECC | 0.28 | 0.82 | 0.31 | 0.80 | 0.42 | 0.70 |
| **YMIPS_4546** | LID | 0.27 | 0.82 | 0.30 | 0.80 | 0.41 | 0.70 |
|  | PeC | 0.30 | 0.83 | 0.34 | 0.80 | 0.44 | 0.71 |
|  | CoEWC | 0.31 | 0.83 | 0.34 | 0.81 | 0.45 | 0.71 |
|  | WDC | 0.30 | 0.83 | 0.34 | 0.81 | 0.45 | 0.71 |
|  | LIDC | **0.42** | **0.86** | **0.46** | **0.84** | **0.56** | **0.76** |
|  |  |  |  |  |  |  |  |

The identification of essential proteins may be considered a two-class classification problem, rather an imbalanced classification problem because there are usually two or three more nonessential proteins than essential proteins in PINs. For example, there were 1167 essential proteins and 3926 nonessential proteins in YDIP_5093 and 1016 essential proteins and 3530 nonessential proteins in YMIPS_4546. Hence, AUC is a suitable measure for estimating the efficiency of classifications between LIDC and other reference methods. The AUCs of LIDC were better than those of all seven other methods in the two PINs (0.73 for YDIP_5093 and 0.67 for YMIPS_4546), and the LIDC exhibited 5.8% and 11.7% improvement, respectively, relative to the best results obtained by the seven other methods. Therefore, the performance of LIDC for this imbalanced two-class classification for identification of essential proteins was best among both PINs, as shown in Table 6.

**Effects of the new integration strategy**

| **Table 6. Comparisons of AUCs between LIDC and the seven other methods in two different PINs.** | | |
| --- | --- | --- |
| **Methods** | **YDIP_5093** | **YMIPS_4546** |
| **DC** | 0.67 | 0.57 |
| **BC** | 0.63 | 0.56 |
| **SoECC** | 0.69 | 0.57 |
| **LID** | 0.69 | 0.56 |
| **PeC** | 0.64 | 0.56 |
| **CoEWC** | 0.65 | 0.56 |
| **WDC** | 0.69 | 0.60 |
| **LIDC** | **0.73** | **0.67** |
|  |  |  |

To assess the effects of the new integration strategy on LIDC in each of the 12 comparisons of YDIP_5093 and YMIPS_4546, we analyzed the relative increasing ratio as a measure for evaluating performance, using LID as the reference method. LID is a topological centrality measure whose performance was poorer than SoECC, a core component in PeC, CoEWC, and WDC, of the 12 comparisons for each PIN, as shown in Figs. 3–6. The relative increasing ratio represented the ratio of the increase of identification method results between two adjacent ranking ranges and the increase of these two adjacent ranking ranges; for example, the relative increasing ratio between the top 1% and top 100 of LID in YDIP_5093 was 31/49 = 0.63, where 31 is the increase in two successive prediction results from the top 1% to top 100, as shown in Figs. 3 and 4, and the denominator 49 is the increase in two successive ranking ranges (top 1% = 51 to top 100). We propose that the relative increasing ratio may describe changes in the performance improvement of methods at each ranking range compared to the former range.

From Fig. 7, there were three relatively steady intervals in the 12 ranking ranges of YDIP_5093 for the relative increasing ratio curve of LIDC compared to that of LID, i.e., from top 100–200, top 300–500, and from top 600 to top 20%. The three intervals comprised eight ranking ranges, indicating that the performance of LIDC may improve steadily in 66.7% of all 12 ranges in YDIP_5093. The results for YMIPS_4546 were similar (Fig. 8). There were also two relative steady intervals for the 12 ranking ranges (from top 5% to top 300 and top 500–600), comprising five ranking ranges to steadily improve the performance of LIDC. Thus, the new integration strategy of LIDC was suitable for decreasing the sensitivity of identification methods for network structures through improving the performance of LIDC in the middle intervals (i.e., from top 300–600) of ranking ranges with decreased impact of LID and increased impact of IDC on LIDC.

Above all, our new strategy may contribute to the enhancement of the LIDC identification method for proteins whose centrality values were located in the middle part of ranking ranges after descending sorting. The second advantage of this new strategy is that it decreases the number of unidentified proteins that have the same centrality scores, improving the performance of LIDC. For example, there were 2705 proteins whose SoECC centrality measures were 0 and 2734 proteins that had 0 values using the PeC multi-information fusion method on the same DIP dataset; in contrast, there were 2430 proteins with 0 values using our LIDC method, a 10% reduction in the number of unidentified proteins. The third advantage of this integration strategy is that LID(*u*) will be changed with different PINs, thereby changing RANK(*u*) and LIDC(*u*). This type of change may increase the flexibility of LIDC when analyzing multiple PINs.

**Fig. 7. Comparison of the relative increasing ratio of essential proteins with various ranking ranges between LIDC and LID in the YDIP_5093 PIN.**

**Fig. 8. Comparison of the relative increasing ratio of essential proteins with various ranking ranges between LIDC and LID in the YMIPS_4546 PIN.**

**Analysis of the differences between LIDC and the seven other methods**

In this section, we described analysis of the differences between prediction results of LIDC and those of the seven other identification methods in order to elucidate the reasons for the improved performance of LIDC. We made a comparison from the top 100 predicted proteins of each method to analyze the relationships among LIDC and the seven other methods, and the number of differences in the top 100 proteins predicted by any two different methods is shown in Table 7. In Table 7, |M-LIDC| indicates the number of proteins identified by method M, not by LIDC, and {LIDC-M} indicates the set of proteins identified by LIDC, not by method M. The |M-LIDC| column shows that the differences between LIDC and the seven other methods were all more than 73% in YDIP_5093 and 61% in YMIPS_4546, indicating that LIDC was a different identification method than the seven other methods. Second, we assessed the proportion of essential proteins in the proteins identified by LIDC and other methods. The remaining columns in Table 7 show how many essential proteins were predicted out of all the different proteins identified by LIDC and other methods. The results showed that the percentage of essential proteins identified by LIDC was higher than that detected by other methods in both PINs consistently. In YDIP_5093, the percentages of essential proteins identified by LIDC for the seven different protein sets were all more than 71%; in contrast, only PeC obtained a percentage of more than 70%. In YMIPS_4546, the lowest percentage of essential proteins identified by LIDC for the seven different protein sets was 65%, while the best percentage obtained by the seven reference methods was 41%, obtained by CoEWC.

Then, we explored the meanings of differences between LIDC and the seven other methods in more detail. There were 92 and 93 different proteins for DC and BC, respectively, compared to LIDC in the top 100 proteins of YDIP_5093. Additionally, the percentages of essential proteins for DC and BC were both 41%, while LIDC obtained 74% and 75% essential proteins. Moreover, in YMIPS_4546, DC and BC both obtained 83 different proteins compared to the top 100 proteins obtained by LIDC, with 14% essential proteins for both DC and BC, versus 64% and 65% for LIDC. Thus, the higher the DC or BC and the lower the LIDC at a single protein, the less likely the protein was to be essential.

According to these results, we propose that LIDC was more effective at predicting essential proteins at the network level.

| **Table 7. Analysis of the differences between LIDC and the seven other methods for**  **prediction of the top 100 proteins.** The results shown at the top of the table are for  the YDIP_5093 PIN, while the results at the bottom of the table are for the YMIPS_  _4546 PIN. | | | | | |
| --- | --- | --- | --- | --- | --- |
| **Methods(M)** | **\|M-LIDC\|** | **Essential proteins in {M-LIDC}** | **Essential proteins in  {LIDC-M}** | **Proportion of Essential  proteins in {M-LIDC}** | **Proportion of Essential  proteins in {LIDC-M}** |
| **DC** | 92 | 38 | 68 | 41% | **74%** |
| **BC** | 93 | 38 | 70 | 41% | **75%** |
| **SoECC** | 75 | 36 | 57 | 48% | **76%** |
| **LID** | 75 | 40 | 55 | 53% | **73%** |
| **PeC** | 77 | 56 | 56 | 73% | **73%** |
| **CoEWC** | 73 | 49 | 52 | 67% | **71%** |
| **WDC** | 73 | 46 | 53 | 63% | **73%** |
| **DC** | 83 | 12 | 55 | 14% | **66%** |
| **BC** | 83 | 12 | 54 | 14% | **65%** |
| **SoECC** | 63 | 8 | 49 | 13% | **78%** |
| **LID** | 61 | 10 | 51 | 16% | **84%** |
| **PeC** | 82 | 33 | 55 | 40% | **67%** |
| **CoEWC** | 74 | 30 | 50 | 41% | **68%** |
| **WDC** | 70 | 10 | 51 | 14% | **73%** |
|  |  |  |  |  |  |

**Assessment of the modularity of proteins predicted by LIDC and four other methods**

Proteins rarely act alone; instead, proteins function in groups, i.e., protein complexes or functional modules, to perform their tasks in biological systems. Hence, protein modularity may be an appropriate approach to evaluate the rationality of essential proteins identified by LIDC in some biological sense. In order to explore the modularity of proteins predicted by LIDC and four other methods (i.e., WDC, CoEWC, PeC, and DC), whose probability to be essential was higher, each of five small PINs were constructed based on the top 100 proteins detected by LIDC and the four reference methods in YDIP_5093 and YMIPS_4546. Each network consisted of the top 100 proteins ranked by LIDC and the four other methods. Their structures are shown in Figs. 9 and 10, respectively, and the probability of the top 100 proteins to be essential was higher. Next, we used clustering with the overlapping neighborhood expansion (ClusterONE) to detect protein modules in these small PINs. In this analysis, we set a *p*-value of less than 0.001 in ClusterONE (*p*-value of less than 0.05 in the original paper) because we expected the protein modules predicted by ClusterONE to possess stronger biological significance, with the module size detected in more than two nodes.

As shown in Table 8, the number of edges (359 by LIDC) was the smallest in the five top 100 PINs of YDIP_5093, and the average number of neighbors and network density, which were 7.18 and 5.14, respectively, were also the smallest for the LIDC PIN. In contrast, the characteristic path length and number of essential proteins in the LIDC PIN were 3.30 and 76, both of which were the largest values for the five top 100 PINs. These results indicated that the top 100 PIN of LIDC was a more sparse PIN than those of the four other methods and contained more essential proteins.

Additionally, ClusterONE detected seven functional modules in the PIN of LIDC, and LIDC yielded the best results in all five top 100 PINs. We found that the most functional modules were in the most sparse top 100 PIN, which was identified by the LIDC method. Moreover, the same results were observed for the five top 100 PINs of YMIPS_4546. Therefore, essential proteins predicted by LIDC possessed more obvious modularity than those identified by the four other methods in YDIP_5093 and YMIPS_4546 PINs.

**Fig. 9. Five subgraphs of the top 100 proteins without isolated nodes predicted through five methods (LIDC, WDC, CoEWC, PeC, and DC) in the YDIP_5093 PIN, in which red nodes are essential proteins and grey nodes are nonessential proteins.**

**Fig. 10. Five subgraphs of the top 100 proteins without isolated nodes predicted through five methods (LIDC, WDC, CoEWC, PeC, and DC) in the YMIPS_4546 PIN, in which red nodes are essential proteins and grey nodes are nonessential proteins.**

| **Table 8. Statistical analyses of five subgraphs of the top 100 proteins predicted by**  **LIDC, WDC, CoEWC, PeC, and DC.** The results shown at the top of the table are for  the YDIP_5093 PIN, while the results at the bottom of the table are for the YMIPS_4546  PIN. | | | | | | | | |
| --- | --- | --- | --- | --- | --- | --- | --- | --- |
| **Methods** | **Number  of Nodes** | **Number of Isolated Nodes** | **Number of  Edges** | **Number**  **of Essential Proteins** | **Characteristic Path Length** | **Average Number  of Neighbors** | **Network Density** | **Number  of Modules** |
| **LIDC** | 100 | 4 | **359** | 76 | **3.30** | 7.18 | **0.07** | **7** |
| **DC** | 100 | 3 | 692 | 46 | 2.22 | 13.84 | 0.14 | 0 |
| **WDC** | 100 | 0 | 484 | 69 | 2.98 | 9.68 | 0.1 | 4 |
| **CoEWC** | 100 | 3 | 499 | 73 | 2.90 | 9.98 | 0.1 | 3 |
| **PeC** | 100 | 3 | 496 | 76 | 3.09 | 9.92 | 0.1 | 5 |
| **LIDC** | 100 | 4 | **257** | 66 | **3.76** | 5.14 | **0.05** | **5** |
| **DC** | 100 | 1 | 671 | 23 | 2.36 | 13.42 | 0.14 | 3 |
| **WDC** | 100 | 0 | 768 | 25 | 2.33 | 15.36 | 0.16 | 4 |
| **CoEWC** | 100 | 8 | 266 | 46 | 3.06 | 5.37 | 0.06 | 2 |
| **PeC** | 100 | 12 | 262 | 44 | 2.64 | 5.24 | 0.05 | 3 |
|  |  |  |  |  |  |  |  |  |

**Conclusions**

In recent decades, there has been a sharp increase in PPI datasets due to the rapid development of experimental technologies, such as affinity purification. These experimental results may produce various PINs, and the identification of essential proteins at the network level has become a major focus of many researchers. Hence, many prediction methods have been proposed based on topological characteristics and the variety of biological information of PINs. However, experiment datasets for many species are still incomplete, leading to false negative and false positive data. On the other hand, capturing the distinct features of essential proteins to improve the performance of identification is still challenging. Research focusing on mechanisms of various biological processes is still in the initial stages. These limits decrease the capabilities of existing prediction methods, making the prediction methods sensitive to the network structure of PINs. In order to overcome the constraints mentioned above, we explored real protein complexes to discover new characteristics of essential proteins and found that the essentiality of proteins may have a close relationship with interactions among proteins in the same clustering. As a result, we propose a new identification, LIDC, as a multi-information fusion method constructed by two information features, i.e., the LID and IDC of essential proteins, which represent new topological characteristics of proteins and in-degree information of protein complexes, respectively, based on the new integration strategy designed in this paper, with the goal of improving performance in multiple PINs.

LIDC was applied to two PINs of *S. cerevisiae*: YDIP_5093 and YMIPS_4546. The experimental results indicated that LIDC significantly outperformed recent classical identification methods, including DC, BC, SoECC, LID, PeC, CoEWC, and WDC. In particular, we achieved 30% improvement in the number of essential proteins identified in YMIPS_4546. Additionally, experimental results also showed that LIDC was quite different from other existing methods and possessed higher accuracy. Moreover, the essential proteins identified by LIDC exhibited stronger modularity, such that the clusters connected by these proteins were also essential for special biological functions, consistent with recent studies . Hence, we concluded that LIDC could achieve better performance of identification methods synthetically through integration of multiple information datasets. In future studies, we will explore new suitable characteristics of essential proteins and examine how to combine multiple information datasets more effectively in order to increase the capability of LIDC.

**Acknowledgments**

We thank the editors and reviewers for valuable suggestions for our manuscript.

**References**

1. Jeong H, Mason SP, Barabasi AL, Oltvai ZN (2001) Lethality and centrality in protein networks. Nature 411: 41-42.

2. Jimenez-Sanchez G, Childs B, Valle D (2001) Human disease genes. Nature 409: 853-855.

3.Gill N, Singh S, Aseri TC (2014) Computational disease gene prioritization: an appraisal. Journal of computational biology : a journal of computational molecular cell biology 21: 456-465.

4. Zhu C, Wu C, Aronow BJ, Jegga AG (2014) Computational approaches for human disease gene prediction and ranking. Advances in experimental medicine and biology 799: 69-84.

5. Giaever G, Chu AM, Ni L, Connelly C, Riles L, et al. (2002) Functional profiling of the Saccharomyces cerevisiae genome. Nature 418: 387-391.

6. Cullen LM, Arndt GM (2005) Genome-wide screening for gene function using RNAi in mammalian cells. Immunol Cell Biol 83: 217-223.

7. Roemer T, Jiang B, Davison J, Ketela T, Veillette K, et al. (2003) Large-scale essential gene identification in Candida albicans and applications to antifungal drug discovery. Molecular Microbiology 50: 167-181.

8. Ren J, Wang J, Li M, Wang H, Liu B (2011) Prediction of Essential Proteins by Integration of PPI Network Topology and Protein Complexes Information. In: Chen J, Wang J, Zelikovsky A, editors. Bioinformatics Research and Applications: Springer Berlin Heidelberg. pp. 12-24.

9. Hsing M, Byler K, Cherkasov A (2008) The use of Gene Ontology terms for predicting highly-connected 'hub' nodes in protein-protein interaction networks. BMC systems biology 2: 80.

10. Li M, Zhang H, Wang JX, Pan Y (2012) A new essential protein discovery method based on the integration of protein-protein interaction and gene expression data. BMC systems biology 6: 15.

11. Yu H, Braun P, Yildirim MA, Lemmens I, Venkatesan K, et al. (2008) High-quality binary protein interaction map of the yeast interactome network. Science 322: 104-110.

12. Zotenko E, Mestre J, O'Leary DP, Przytycka TM (2008) Why do hubs in the yeast protein interaction network tend to be essential: reexamining the connection between the network topology and essentiality. PLoS computational biology 4: e1000140.

13. Wang H, Kakaradov B, Collins SR, Karotki L, Fiedler D, et al. (2009) A complex-based reconstruction of the Saccharomyces cerevisiae interactome. Molecular & cellular proteomics : MCP 8: 1361-1381.

14. Ryan CJ, Krogan NJ, Cunningham P, Cagney G (2013) All or nothing: protein complexes flip essentiality between distantly related eukaryotes. Genome Biol Evol 5: 1049-1059.

15. Fraser HB, Plotkin JB (2007) Using protein complexes to predict phenotypic effects of gene mutation. Genome Biol 8: R252.

16. Lage K, Karlberg EO, Storling ZM, Olason PI, Pedersen AG, et al. (2007) A human phenome-interactome network of protein complexes implicated in genetic disorders. Nat Biotechnol 25: 309-316.

17. Oti M, Brunner HG (2007) The modular nature of genetic diseases. Clinical genetics 71: 1-11.

18. Joy MP, Brock A, Ingber DE, Huang S (2005) High-betweenness proteins in the yeast protein interaction network. Journal of biomedicine & biotechnology 2005: 96-103.

19. Jianxin W, Min L, Huan W, Yi P (2012) Identification of Essential Proteins Based on Edge Clustering Coefficient. Computational Biology and Bioinformatics, IEEE/ACM Transactions on 9: 1070-1080.

20. Zhang X, Xu J, Xiao WX (2013) A new method for the discovery of essential proteins. PloS one 8: e58763.

21. Xiwei T, Jianxin W, Jiancheng Z, Yi P (2014) Predicting Essential Proteins Based on Weighted Degree Centrality. IEEE/ACM Transactions on Computational Biology and Bioinformatics 11: 407-418.

22. Li M, Wang J, Chen X, Wang H, Pan Y (2011) A local average connectivity-based method for identifying essential proteins from the network level. Computational biology and chemistry 35: 143-150.

23. Li M, Zheng R, Zhang H, Wang J, Pan Y (2014) Effective identification of essential proteins based on priori knowledge, network topology and gene expressions. Methods 10.1016/j.ymeth.2014.02.016.

24. Pu S, Wong J, Turner B, Cho E, Wodak SJ (2009) Up-to-date catalogues of yeast protein complexes. Nucleic acids research 37: 825-831.

25. Xenarios I, Rice DW, Salwinski L, Baron MK, Marcotte EM, et al. (2000) DIP: the Database of Interacting Proteins. Nucleic acids research 28: 289-291.

26. Mewes HW, Frishman D, Mayer KFX, Münsterkötter M, Noubibou O, et al. (2006) MIPS: analysis and annotation of proteins from whole genomes in 2005. Nucleic acids research 34: D169-D172.

27. Cherry JM, Adler C, Ball C, Chervitz SA, Dwight SS, et al. (1998) SGD: Saccharomyces Genome Database. Nucleic acids research 26: 73-79.

28. Zhang R, Lin Y (2009) DEG 5.0, a database of essential genes in both prokaryotes and eukaryotes. Nucleic acids research 37: D455-D458.

29. Winzeler EA, Shoemaker DD, Astromoff A, Liang H, Anderson K, et al. (1999) Functional Characterization of the S. cerevisiae Genome by Gene Deletion and Parallel Analysis. Science 285: 901-906.

30. Friedel CC, Krumsiek J, Zimmer R (2009) Bootstrapping the Interactome: Unsupervised Identification of Protein Complexes in Yeast. Journal of Computational Biology 16: 971-987.

31. Aloy P, Böttcher B, Ceulemans H, Leutwein C, Mellwig C, et al. (2004) Structure-Based Assembly of Protein Complexes in Yeast. Science 303: 2026-2029.

32. Pu S, Vlasblom J, Emili A, Greenblatt J, Wodak SJ (2007) Identifying functional modules in the physical interactome of Saccharomyces cerevisiae. Proteomics 7: 944-960.

33. Mason SJ, Graham NE (2002) Areas beneath the relative operating characteristics (ROC) and relative operating levels (ROL) curves: Statistical significance and interpretation. Quarterly Journal of the Royal Meteorological Society 128: 2145-2166.

34. Nepusz T, Yu H, Paccanaro A (2012) Detecting overlapping protein complexes in protein-protein interaction networks. Nature methods 9: 471-472.

35. Wuchty S (2014) Controllability in protein interaction networks. Proceedings of the National Academy of Sciences 111: 7156-7160.

**Figure Legends**

**Fig. 1. Protein YJL143W (red) is a no-star node with its five neighbors.** VNB(u) = VNB_ISO(u)∪VNB_INT(u), u = YJL143W, VNB_INT(u) = {YNR017W,YJR045C, YNL131W,YMR203W}, VNB_ISO(u) = {YJL064W}. Five blue long dashes represent the adjacent edges between YJL143W and its neighbors, and the two black solid lines represent interactive edges among interactive neighbors colored by purple. Green nodes are isolated neighbors, such as protein YJL064W. Protein YJL143W is an essential protein.

**Fig. 2. Protein YDL171C (red) is a star node with its five neighbors.** The blue long dashes are the adjacent edges between the source node YDL171C and its green neighbors, which are completely isolated, meaning they will be isolated points in the adjacency subgraph of u if these adjacent edges can be ignored. Protein YDL171C is a nonessential protein.

**Fig. 3. Comparison of the number of essential proteins from the top 100–600 identified by LIDC and seven other prediction measures in the YDIP_5093 PIN.**

**Fig. 4. Comparison of the number of essential proteins from the top 1%–25% identified by LIDC and seven other prediction measures in the YDIP_5093 PIN.**

**Fig. 5. Comparison of the number of essential proteins from the top 100–600 identified by LIDC and seven other prediction measures in the YMIPS_4546 PIN.**

**Fig. 6. Comparison of the number of essential proteins from the top 1%–25% identified by LIDC and seven other prediction measures in the YMIPS_4546 PIN.**

**Fig. 7. Comparison of the relative increasing ratio of essential proteins with various ranking ranges between LIDC and LID in the YDIP_5093 PIN.** **.**

**Fig. 8. Comparison of the relative increasing ratio of essential proteins with various ranking ranges between LIDC and LID in the YMIPS_4546 PIN.** **.**

**Fig. 9. Five subgraphs of the top 100 proteins without isolated nodes predicted through five methods (LIDC, WDC, CoEWC, PeC, and DC) in the YDIP_5093 PIN, in which red nodes are essential proteins and grey nodes are nonessential proteins.**

**Fig. 10. Five subgraphs of the top 100 proteins without isolated nodes predicted through five methods (LIDC, WDC, CoEWC, PeC, and DC) in the YMIPS_4546 PIN, in which red nodes are essential proteins and grey nodes are nonessential proteins.**

**Table Legends**

**Table 1. The two yeast protein-protein interaction networks obtained from two PPI datasets (DIP and MIPS).**

**Table 2. The global characteristics of two yeast protein interaction networks.**

**Table 3. The local characteristics of two yeast protein interaction networks.**

**Table 4. Statistical analyses of improvement according to comparisons between LIDC and the best results of the seven other methods in each ranking range.** The results at the top of the table are for the YDIP_5093 PIN, and the rest are for the YMIPS_4546 PIN.

**Table 5. Comparison of the sensitivity (SN), specificity (SP), positive predictive value (PPV), negative predictive value (NPV), F-measure (F), and accuracy (ACC) between LIDC and the seven other prediction methods for the top 20% of proteins in two different PINs.**

**Table 6. Comparisons of AUCs between LIDC and the seven other methods in two different PINs.**

**Table 7. Analysis of the differences between LIDC and the seven other methods for prediction of the top 100 proteins.** The results shown at the top of the table are for the YDIP_5093 PIN, while the results at the bottom of the table are for the YMIPS_4546 PIN.

**Table 8. Statistical analyses of five subgraphs of the top 100 proteins predicted by LIDC, WDC, CoEWC, PeC, and DC.** The results shown at the top of the table are for the YDIP_5093 PIN, while the results at the bottom of the table are for the YMIPS_4546 PIN.

Thank you again and best regards.

Yours sincerely,

Yi Qi
